# Silk fibroin-based scaffolds for tissue engineering

**DOI:** 10.3389/fbioe.2024.1381838

**Published:** 2024-04-25

**Authors:** Li Ma, Wenyuan Dong, Enping Lai, Jiamian Wang

**Affiliations:** ^1^ National Innovation Center for Advanced Medical Devices, Shenzhen, China; ^2^ College of Biological and Chemical Engineering, Guangxi University of Science and Technology, Liuzhou, China

**Keywords:** silk fibroin, biomaterials, scaffolds, tissue engineering, regeneration

## Abstract

Silk fibroin is an important natural fibrous protein with excellent prospects for tissue engineering applications. With profound studies in recent years, its potential in tissue repair has been developed. A growing body of literature has investigated various fabricating methods of silk fibroin and their application in tissue repair. The purpose of this paper is to trace the latest developments of SF-based scaffolds for tissue engineering. In this review, we first presented the primary and secondary structures of silk fibroin. The processing methods of SF scaffolds were then summarized. Lastly, we examined the contribution of new studies applying SF as scaffolds in tissue regeneration applications. Overall, this review showed the latest progress in the fabrication and utilization of silk fibroin-based scaffolds.

## 1 Introduction

Tissue engineering is a biomedical engineering discipline that combines living cells, suitable biochemical (e.g., growth factors) and physical (e.g., cyclic mechanical loading) factors, and biocompatible materials in rebuilding, preserving, improving, or replacing different types of biological tissues. It usually involves implanting tissue construction into the body to fix an injury or restore a failing function of the organ (Berthiaume et al., 2011). A suitable material for tissue regeneration should conform to the following: biocompatible, nontoxic, controlled biodegradability, proper architecture, mechanical properties, antibacterial properties (Bhattacharjee et al., 2017; Xie et al., 2021). To date, synthetic materials such as metal, ceramics, bioactive glass, polymers have been widely used for tissue regeneration. Compared to synthetic materials, natural polymers offer better compatibility, which is a prerequisite for the qualification of artificial implants. Also, their inherent bioactivity rendered positive attributes towards graft regeneration. Hence, natural ECM proteins are prevalently decorated with cell-binding sites, which assist in cellular adhesion and migration (Silk biomaterials for vascular tissue engineering applications). From a clinical perspective, scaffolds for tissue engineering are a combination of mechanical, chemical, and biological properties (Ma et al., 2003; Suesca et al., 2017; Xu et al., 2017). Therefore, in tissue engineering scaffold design, the properties of biomaterials should be fully focused and regulated to satisfy the clinical requirements (Kundu et al., 2013).

Due to its outstanding mechanical characteristics and sheen, silk has been used in the textile industry for over a millennium. It is biosynthesized in epithelial cells by more than 200,000 arthropods such as silkworms, spiders, lacewing, glowworms, and mites (Craig, 1997; von Byern et al., 2019). The most characterized silk was usually produced by *B. mori* (*Bombyx mori*)(Sun J. et al., 2021). The natural biopolymer known as silk fibroin (SF), which is derived from *B. mori* silk, is widely accessible and has been employed for many years as sutures in the human body. It was exploited in many biomedical science areas due to the constant development of its processing technology and outstanding properties, including excellent mechanical properties, biocompatibility, slow degradation, and sterilizability (Vepari and Kaplan, 2007; Kundu et al., 2013). It has been proven to be a promising ingredient for biomedical application. Tissue-engineered scaffolds with highly repeatable shapes, including sponges, films, fibers, and hydrogels, can be produced from SF (Rockwood et al., 2011). It is possible to combine silk proteins with other materials to enhance or achieve specific characteristics, such as biomedical properties including cell adhesion, and biocompatibility (Yang et al., 2014; Liu et al., 2019; Ullah and Chen, 2020). SF-based scaffolds, imitating the extracellular matrix of the native tissue, serve as a physical structure to interact with cells and vessels and supported newly formed tissues. After implantation, cells and vessels migrate and grow into holes in scaffolds. With granulation tissue formation, the scaffold degrades continuously and is replaced completely by reformed tissue.

A large number of published studies (e.g., Hodgkinson, 2014, Pg and Bbmab, 2021) have reviewed SF-based scaffolds in bone, skin, and nerve repair (Hodgkinson, 2014; Gupta and Mandal, 2021). However, given the blooming attraction of silk in tissue engineering and technology areas on the fabrication of silk constructs, a more thorough and current review is necessary. This review summarized the most recent research development in SF-based scaffolds for tissue engineering involving skin, bone, blood vessels, cartilage, ligaments, tendons, and nerves. Particular attention is given here to some of the clinical and marketable advances in SF-based scaffolds in recent years, which may provide some guidance for laboratory research.

## 2 Physicochemical properties of SF as biomaterials

### 2.1 Primary structure

**FIGURE 1:**
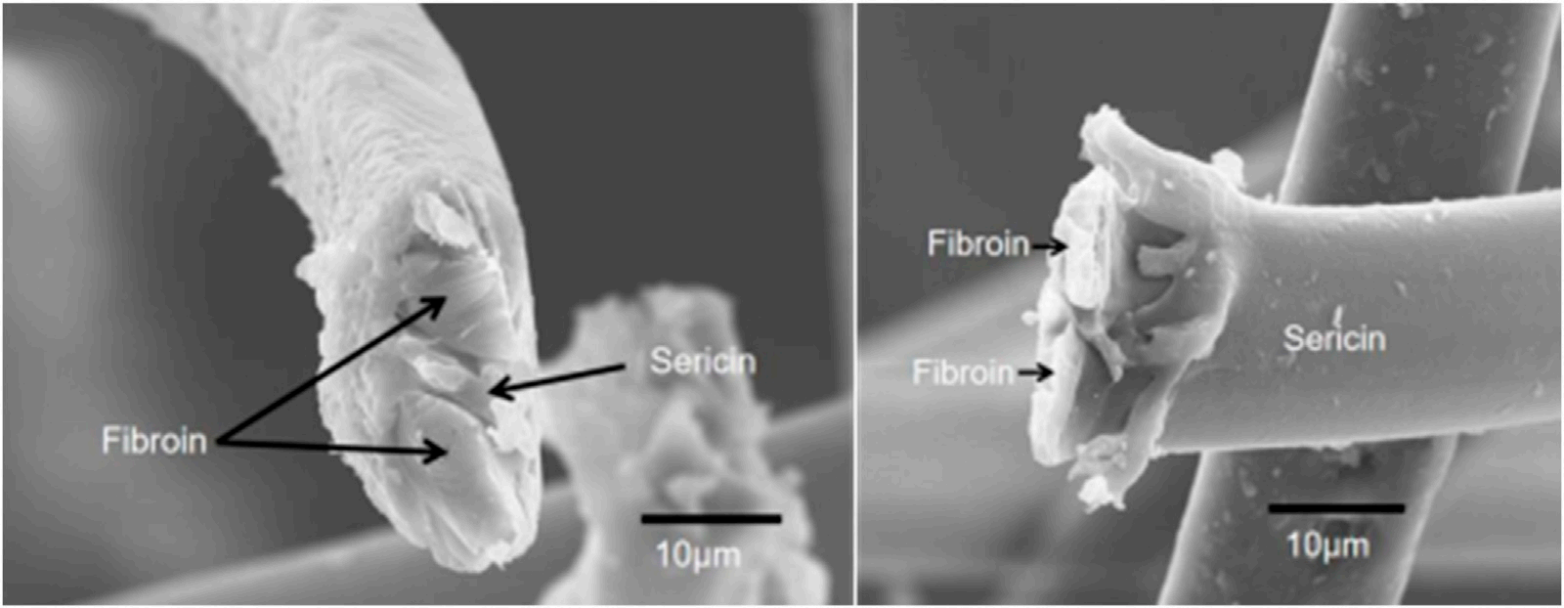
Structure of silk.

Silk cocoon is a single fiber about 700–1,500 m in length and 10–16 μm in diameter. It should be noted that the diameter of this fiber varies greatly in different locations. The diameter of the coarsest place was 2–3 times that of the thinnest place in a fiber. Additionally, the diameter and mechanical characteristics of silk significantly varied in different intraspecies and intraindividual (Zhao et al., 2007). As shown in Figure 1, the single fiber (prepared by our group) consists of three main parts, the outer layer with a sericin coating and two inner fibroins with irregular shapes, concordant with the previous report (Poza et al., 2002).

Sericin coating, also known as silk gum, accounted for 25%–30% of raw silk. Sericin was a group of serine-rich glycoproteins whose molecular weight ranged from 20 to 400 kDa, and it was produced in the middle gland of *B. mori* (Kunz et al., 2016). It was highly hydrophilic and composed of 18 amino acids (Sothornvit et al., 2010). The controversy over the scientific evidence for the biosafety of silk gum has continued unabated over the years. Sericin glue-like proteins were conjectured to induce immunogenicity and hypersensitivity (Altman et al., 2003). Contradictory findings about the function of sericin have been reported in more recent literature (Ahsan et al., 2018; Xiaohalati et al., 2024). To date, the biosafety of sericin has not been elucidated clearly.

### 2.2 Secondary structure

Secondary structures of SF, including silk I, silk II, and silk III, determined the properties of biomaterials. Silk I and silk II were the dominant crystalline structures of SF. Silk I, a metastable crystalline structure with bound water molecules, was a liquid that contains α-helix and even random coil structures (Wohlrab et al., 2012). When exposed to methanol or potassium chloride, it could convert into a silk II structure. Silk II was an unstable and insoluble state. It consisted of antiparallel β-sheets that exhibited greater structural compactness and stability in aqueous conditions. Concurrently, this configuration exhibited stronger mechanical characteristics compared to α-helices and random coils. At the air-water interface, Silk III was a three-fold helical secondary structure that distinguished the hydrophilic serine residues from the hydrophobic alanine residues (He et al., 1999).

## 3 Processing of SF biomaterials

### 3.1 Extraction of silk fibroin

**FIGURE 2:**
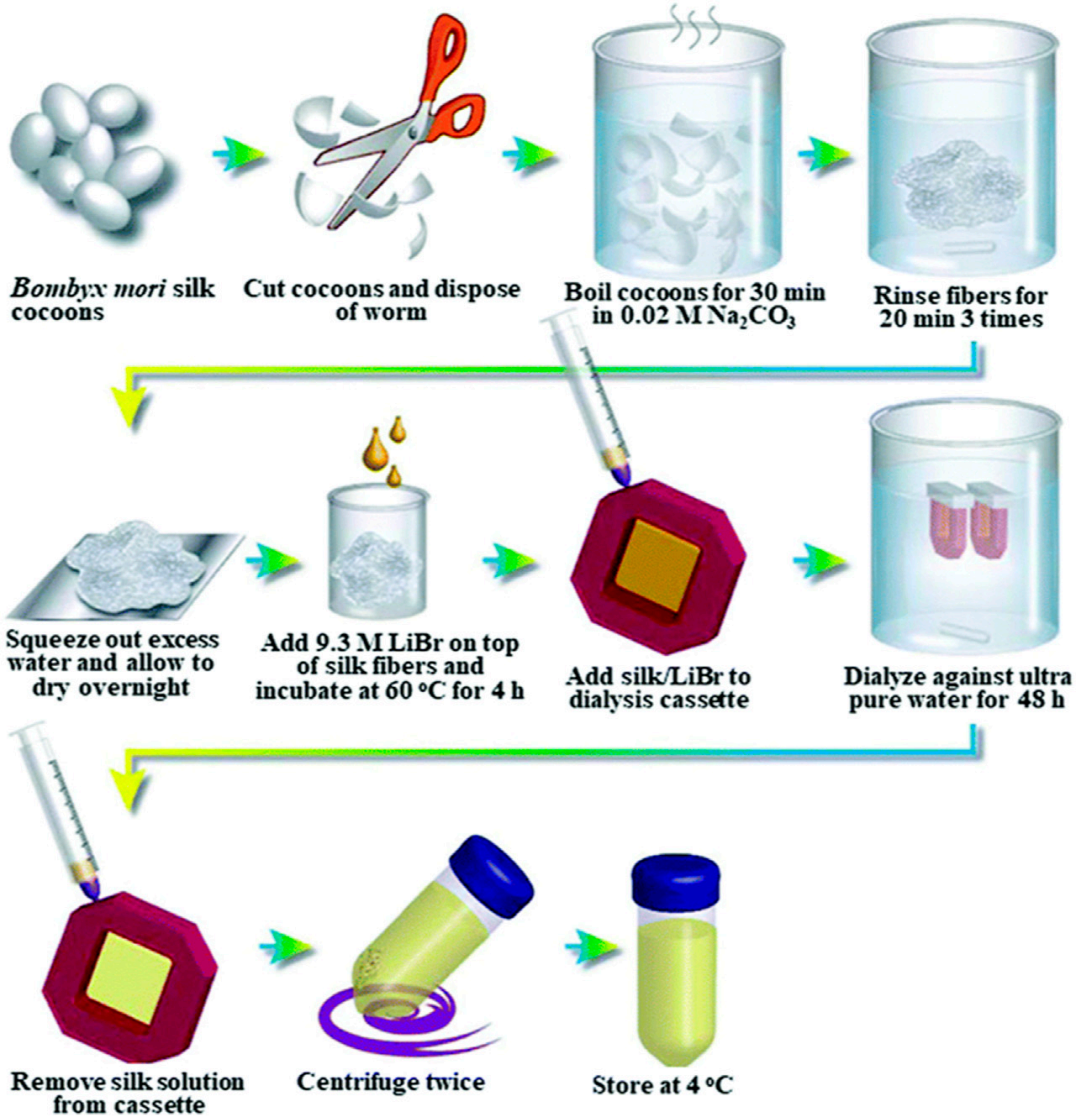
General steps of silk fibroin extraction. Reprinted with permission of ref (Rockwood et al., 2011). Copyright 2011, Springer Nature.

To prepare SF-based biomaterials of different morphology, the SF had to be extracted from the cocoons and dissolved into an aqueous solution in the first place. Generally, the first step of extraction was degumming. It reduced the toxicity caused by sericin. The following process normally was dissolution and dialysis. A comprehensive summary of various aqueous or organic solvent-processing techniques for SF extraction from *B. mori* cocoons, as well as the procedures involved in manufacturing hydrogels, tubes, sponges, composites, fibers, microspheres, and thin films, was provided by Rockwood et al. (Rockwood et al., 2011). Here are some general steps (as shown in Figure 2).

#### 3.1.1 Degumming

The most common protocol for removing the immunogenic sericin coating was to repeat boiling silk in 0.02 M sodium carbonate (Na_2_CO_3_) for 30 min, a total of 3 times. Other chemicals such as Marseille soap, soda, ethylenediamine, tartaric acid, H_2_O_2_, alkaline solution, and alkali were used for degumming of cocoons (Gai et al., 2020; Anand et al., 2021). However, Na_2_CO_3_ was still more effective and resulted in a higher crystallinity index (Kim et al., 2017). Additionally, the enzymatic degumming process was also an attractive method because silk yarn treated with enzyme had a good texture and improved gloss (Sampaio et al., 2015; Wang et al., 2019; DeBari et al., 2021). For example, the cocoonase is a mild enzyme that can retain the original color, smoothness, and shine of silk (Rodbumrer et al., 2012; Unajak et al., 2015; Anand et al., 2021). Recently, Liu et al. investigated the efficiency of several neutral proteases in the degumming of filipin and their effects on the molecular weight of SF. It was discovered that neutral protease was able to both effectively remove silk gum and preserve the integrity of SF. Furthermore, compared to the SF generated by sodium carbonate degumming, the molecular weight of the resultant SF was noticeably higher (Liu et al., 2023).

#### 3.1.2 Dissolution

Degummed silk fibers were often dissolved first and then reshaped to various morphologies for different applications. Silk fibers might be dissolved in either aqueous or organic solvent (e.g., 1,1,1,3,3,3-hexafluoro-2-propanol (HFIP), formic acid). The aqueous solvent includes Ajisawa’s ternary solvent (CaCl_2_/ethanol/water), NaSCN/LiSCN solution (Saturated aqueous solution), LiBr (9.3 M) or LiBr alcohol-H_2_O solution, and Nitrate solution. The SF solution dissolved in these solvents can be used directly without dialysis (Wang et al., 2020). Aqueous SF solutions could be lyophilized and then redissolved in HFIP for long-term storage. However, it is exceedingly difficult to employ for large manufacturing of SF due to its toxicity, high expense, and strong corrosiveness (Lozano-Pérez et al., 2015).

### 3.2 Processing of SF scaffolds

**FIGURE 3:**
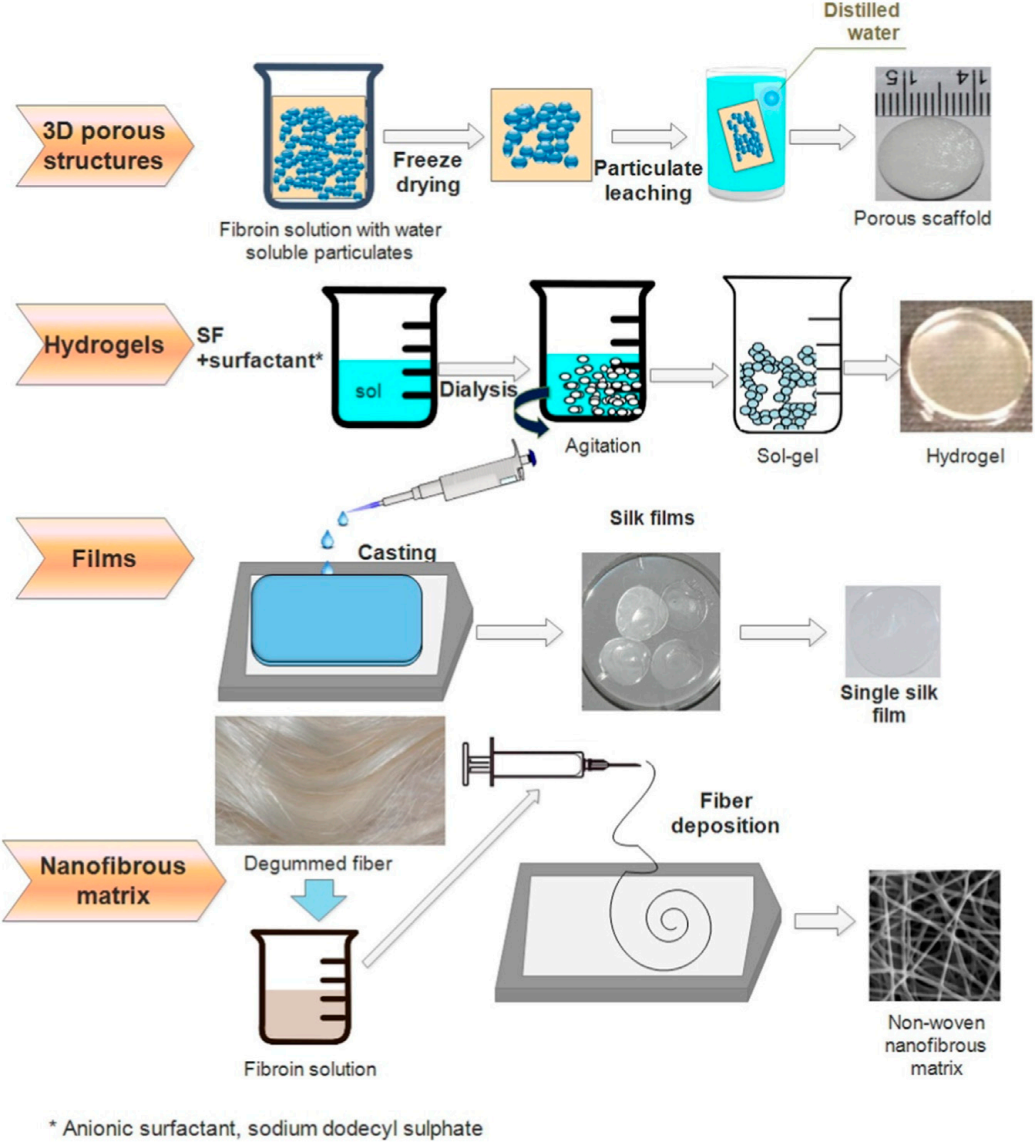
Illustrations of four typical SF scaffolds fabrications. Reprinted with permission of ref (Bhattacharjee et al., 2017). Copyright 2017, Elsevier.

Processing methods determined the physical structure and morphology of scaffolds and thereby influenced the clinical effects. Different components, parameters, and post-treatment methods were introduced to modify the pore size, alignment, and porosity of the porous 3D SF-based scaffold. In recent years, with the development of technologies and the improvement of fabrication equipment, the processing methods of SF were constantly being optimized to meet more requirements. Figure 3 illustrates the process of fabricating four typical SF scaffolds. In this section, the most widely used techniques for SF-based scaffolds, including electrospinning, freeze-drying, solvent casting, gas foaming, particulate leaching, and 3D printing, have been summarized.

#### 3.2.1 Electrospinning

Electrospinning is a spinning technique using an electrical input to produce fibers from a polymer solution. Electrospinning enabled the fabrication of non-woven mats with previously unobtainable nanometric features in terms of pore size and structure (Belbéoch et al., 2021). In comparison to conventional techniques, the nonwoven nanofibrous mats produced by this technique were closer to the extracellular matrix (Chen et al., 2023). The detailed process can be found in the reviews by Pham et al., Zhang et al., Bhardwaj et al., and Garg et al. before 2010 (Pham et al., 2006; Zhang et al., 2009; Bhardwaj and Kundu, 2010; Garg and Bowlin, 2011). To be simplified, the SF solution was poured into a syringe of a spinning machine in the first and the syringe was fixed at some distance and angle. The applied voltage could be adjusted between 10–35 kV. The flow rate was then set between 0.2–4.0 mL/h. A Taylor cone formed as a result of Coulombic forces at the droplet’s interphase when the strong electric field caused an increase in electrostatic repulsion. Whipping instabilities caused the charged jet to continue to extend as it solidified since the solvent was evaporating. Ultimately, an oppositely charged collector received the dry fiber (Humenik et al., 2018). The morphology and diameter of SF fibers were affected by the spinning dope (nanostructures, concentration, viscosity, and solvent) and processing parameters (voltage, flow rate, temperature, and distance between capillary tip and collector).

#### 3.2.2 Solvent casting particulate leaching

Solvent casting particle leaching is a porous scaffold-producing technology. Usually started with solution polymers into a volatile organic solvent, then water-soluble salt particles were added into the mixture. The following step was casting the mixture into a three-dimensional scaffold mold. In the end, the scaffolds were soaked in a solvent that dissolves the particles but not the polymer. Particles in the scaffold were dissolved and left pores in the original place (Grinberg et al., 2010). Salt particles, sugar, glucose, paraffin, gelatin, and ammonium chloride could be introduced to create pores or channels (Raeisdasteh et al., 2017). The porous morphology can be controlled by the shape, size and quantity of the added pore-forming agent (Plikk et al., 2009). The number of articles involved in this method has grown with an explosion trend since 2000. The technology has been used to manufacture silk-based membranes (Jabbari et al., 2019), hydrogels (Srisawasdi et al., 2015), and sponges (Lee et al., 2018; Park et al., 2018). Casting solvents played an important role in the properties of the SF-based scaffold by altering the β-sheet content. The fibroin degradation rate was significantly affected by the casting solvents (trifluoroacetic acid vs. water and formic acid)(Park et al., 2018).

#### 3.2.3 Freeze-drying

Freeze-drying/lyophilization is one of the most common methods for scaffold fabrication. The aqueous silk solution was poured into a mold and then placed in an ultralow-temperature freezer to cool the material under its triple point (Gaidhani et al., 2015). In the second phase, frozen materials together with the mold were transferred to a freeze dryer. Approximately 95% of the water in the material could be removed in this phase. The rest ionic bonded water was removed during the second drying phase. Normally, the temperature is higher than the primary drying phase to break the bonds between the material and the water molecules. In the freeze-drying process, water molecules directly sublimated from ice to steam. When all water molecules were sublimated, a porous scaffold structure was formed. The porosity and pore size of the scaffolds could be affected by polymer solution parameters, including concentration, viscosity, and the amount of aqueous phase dispersed in the system (Janik and Marzec, 2015). Higher levels of polymers in the continuous phase led to lower porosity and smaller pores (Grinberg et al., 2010). Consequently, the size and structure of the micropores could be regulated by controlling the polymer concentration or viscosity. One of the newest attempts at freeze-drying was a facile two-step freeze-drying technology. After being diluted, the carbodiimide-activated SF solution was added to the porous SF scaffolds that had already been constructed. Subsequent liquid nitrogen freezing and lyophilization, the solution then formed into a micro/nanofibrous network inside the porous scaffolds’ pore spaces. Fibers of the network served as topographic cues in the 3D scaffold for cell attachment, proliferation and migration (Li et al., 2016).

#### 3.2.4 Gas foaming

Gas foaming is a method of making synthetic matrices by avoiding solvents to produce pores. The advantages of this method were that either hydrophilic or hydrophobic biopolymers could blend with the polymer matrix (Costantini and Barbetta, 2018). Whenever the polymer was saturated with gas, a sudden drop in pressure caused a thermodynamic instability in the polymer/gas solution, which resulted in the initiation and expansion of cells/pores (Kramschuster and Turng, 2012). It typically includes three basic steps: 1) polymer/gas solution formation, 2) gas bubble nucleation, 3) gas bubble growth and volume expansion (Jiang et al., 2015). Maniglio et al. adopted a single-step method to prepare a fibroin scaffold by applying N_2_O as the foaming agent. In this approach, pore dimensions were directly correlated with gas pressure and inversely correlated with the initial protein concentration (Maniglio et al., 2018). Rao et al. fabricated polylactic acid (PLA)/SF nanofibrous sponge scaffolds by combination electrospinning and gas-foaming technique (Rao et al., 2019). It was the first expanded 3D nanofiber-sponge scaffolds with orientation and porosity by these combined techniques. Chen et al. manufactured Poly (l-lactide-co-caprolactone) (PLCL)/SF nanofibrous scaffolds and then soaked them in a sodium borohydride solution to create three-dimensional scaffolds by applying the techniques of freeze-drying and *in situ* gas foaming. Following 20 min of foaming treatment, the porosity of the scaffolds rose from 72% to 94%, according to the results (Chen et al., 2021). One step further, Hajiabbas et al. combined electrospinning, *in situ* gas foaming, *in situ* crosslinking, and freeze-drying methods to prepare an SF-based composite scaffold. They found that the physicochemical properties of scaffolds were greatly influenced by the structure and degree of crosslinking (Hajiabbas et al., 2020).

#### 3.2.5 3D printing

3D printing is a promising technology to recreate customized and functional materials. The main factors, including rheology, swelling ratio, and surface tension, should be carefully considered when the scaffold was prepared by 3D printing (Hölzl et al., 2016). Silk fibroin, with its processability and tunable mechanical properties, holds great potential for 3D printing of complex tissue scaffolds that mimic the native tissue microenvironment, thereby promoting cell growth and tissue regeneration. The rheological property of SF bio-ink could be regulated by the purification and concentrated process of SF(Wang et al., 2019). However, SF solutions were usually hard to print individually due to their low viscosity and inappropriate rheology (Sun et al., 2012; Lee et al., 2018). With the SF solution’s excellent physical and chemical properties, SF bio-ink was combined with other biomaterials to fulfill different requirements of 3D printing, such as printability, mechanical properties, shape fidelity, and cell viability (Chimene et al., 2016). Gelatin and hydroxypropyl methylcellulose (HPMC) were suitable to adjust the printability and mechanical properties of SF-based bio-ink (Xiong et al., 2017). The combination of SF and gelatin significantly balanced mechanical properties and degradation rate (Shi et al., 2017). For instance, Das et al. developed an SF-gelatin scaffold by 3D bioprinting with satisfied mechanical property (Das et al., 2015). It was benefit for the growth of wrapped mesenchymal progenitor cells with the degradation of this scaffold. Dong et al. adopted a two-step procedure to print SF. In the first step, the SF was mixed with HPMC aqueous solutions. Subsequently, the combination was printed directly onto the pre-established architecture, exhibiting an amazing thixotropic characteristic derived from “the second network.” After the bioprinted SF/HPMC was ripened in ethanol, it presented significant enhanced mechanical properties caused by improved β-sheet domain (Dong et al., 2019). Compaan et al. also designed a two-step process to promote the gelation of SF as a 3D printing component. Calcium alginate was blended with SF to accelerate gelation, and horseradish peroxidase was introduced to catalyze covalent cross-linking. This two-step process allowed 3D bioprinting of cell-loaded SF scaffolds suitable for long-term cell culture (Compaan et al., 2017). Kim et al. introduced glycidyl methacrylate when preparing the SF solution. With the assistance of this processed SF bio-ink, extremely complex organ architectures could be created with good structural stability and consistent biocompatibility (Kim et al., 2018). More information about the requirements, characteristics, and processabilities of SF bio-ink could be found in some profound reviews (Wang et al., 2019; Agostinacchio et al., 2021).

## 4 Applications

### 4.1 Skin tissue regeneration

*In vitro* studies revealed that SF material has the same biosafety performance as commercial graft gelfoam^®^ and ologen^®^ in tissue repair (Bhardwaj et al., 2015). Exceptional mechanical properties and slower biodegradability of SF made it a remarkable component for artificial substitute (Sultan et al., 2018). In the meanwhile, SF scaffolds improved cell motility and expressions of extracellular matrix production-related genes (Hashimoto et al., 2020).

SF scaffolds loaded with cytokines, bioactive components, cells, and tissues could not only provide physical support also act as a delivery system for wound care (Bazrafshan et al., 2014; Wöltje et al., 2018; Li et al., 2019; Lehmann et al., 2022). With the scientific work on wound healing, researchers developed an SF-based fibrous scaffold to deliver stem cells in burn wound rats. In this study, SF containing stem cells offered a large surface area, cellular behavior guiding, and scar reduction while closely imitating the biophysical and biochemical characteristics of the native extracellular matrix (ECM)(Huang et al., 2019). It can be inferred from the study that the architectural features of SF provided a bio-mimetic system for the differentiation of stem cells in advance. Another study also demonstrated that collagen synthesis and skin re-epithelization can be significantly accelerated by using an SF nanofibrous scaffold loaded with mesenchymal stem cells (MSCs) and epidermal stem cells; in addition, the histological features and skin appendages of the reconstructed skins resembled those of normal rat skin (Xie et al., 2016). Also, collagen/SF hybrid scaffolds loaded with bone mesenchymal stem cells had excellent skin affinity, air and water permeability (Cui et al., 2020).

In recent years, more and more research has been devoted to developing SF scaffolds with antibacterial properties for wound dressings (Babu et al., 2018; Tariq et al., 2021; Chizari et al., 2022; Dong et al., 2022; Dehghani et al., 2023; Li et al., 2024). Interestingly, Sen et al. immobilized SF into the surface of polyurethane (PU) scaffolds. The growth of K. pneumonia, bacteria found in wound infection, could be inhibited by SF at 8 mg/mL (Sen et al., 2020). SF/Poly (l-lactide-co-caprolactone) (PLCL) nanofibrous membrane loaded with oregano essential oil (OEO) had been studied for wound healing. In this study, a relatively high amount of OEO was loaded into a SF matrix relying on physical interaction through electrospinning. Both *in vitro* and *in vivo* results showed that the synchronization of SF membranes and OEO’s bioactivity had a beneficial impact on the healing process (Huang et al., 2020). However, some antibacterial compounds loaded in SF dressings may cause side effects. It is still a challenge to balance the biocompatibility and antibacterial properties. For example, zinc oxide (ZO) could be applied in the hyaluronic acid/SF/ZO nanofibrous wound dressing to improve the antibacterial property, nevertheless, high concentrations of ZO (>3 wt%) were harmful to the cells, according to *in vitro* cytotoxicity experiments (Hadisi et al., 2020). Zhang et al. found that doping Pluronic polymers in SF films optimized their mechanical properties, hydrophilicity, and light transmission. The obtained SF scaffold can be used to encapsulate antimicrobial agents (e.g., curcumin, Ag nanoparticles, and the antimicrobial peptide KR-12, etc.). Additionally, *in vitro* tests revealed that this film had the ability to continuously release antimicrobial agents, killing bacteria. *In vivo* tests revealed that, independent of the antimicrobial agents loaded within, the film not only eliminated methicillin-resistant *Staphylococcus aureus* from the wound area and reduced inflammation, but also aided in angiogenesis and re-epithelialization, hastening the healing process for infected wounds (Zhang et al., 2024). Genetic engineering was applied to develop SF-based wound healing materials with better performance (Wöltje et al., 2018). One study developed an SF-based film with transgenic worms, which overexpressed the arginine-glycine-aspartic acid (RGD) sequence. The results confirmed that the transgenic SF-based film has more profound effects on wound closure, granulation formation, and cell proliferation than conventional ones (Baba et al., 2019). Genetically modified SF-based scaffolds allow the production of low-cost artificial skin with additional functionality, which makes minimal scarring possible. Moreover, SF is an FDA-approved material, and a number of cosmetic and medical applications (Fibroheal™ Ag Wound Dressing) based on SF are currently available. Four of the clinical trials already available are on skin tissue, with two of them nearing completion. SF scaffolds are promising for skin tissue engineering, especially for wound dressings and skin grafts, due to their superior biocompatibility and bioactivity.

### 4.2 Bone tissue regeneration

For bone tissue regeneration, SF is a good option due to its outstanding biocompatibility, favorable cell attachment, growth, differentiation, and migration, as well as its capacity to promote osteogenesis and oxygen transport (Kuboyama et al., 2013; Melke et al., 2016; Choi et al., 2018; Ghanbari et al., 2023; Li et al., 2023). Maraldi et al., 2011 assessed the mineralization ability of amniotic fluid stem cells cultured in different porous scaffolds, including collagen, poly-D, L-lactic acid (PDLLA), and SF. The results showed that mineralization of stem cells was enhanced substantially on the SF scaffolds compared with collagen and PDLLA scaffolds, which means silk scaffold was more favorable for osteogenic differentiation (Li et al., 2019). *In vitro* osteogenic development of human adipose-derived mesenchymal stem cells (hASCs) might be greatly enhanced by SF scaffolds. In a mouse critical-sized calvarial defect repair experiment, Riccio et al. found that the SF scaffold could repair calvarial defects whether or not human stem cells were pre-seeded in the SF scaffold, even though the higher bone quantity were found in the SF scaffold group which pre-implanted with stem cells (Riccio et al., 2012). Wu et al. recently manufactured PLLA/SF composite nanofiber mesh via electrospinning, and coated osteoblast-derived extracellular matrix (O-ECM) on the nanofiber scaffold. The novel nanofiber scaffold (O-ECM/PLLA/SF) has been shown, through *in vitro* testing, to significantly enhance the osteogenic differentiation of cultivated stem cells (Wu et al., 2022).

SF-hydroxyapatite (HAp) nanocomposite has shown greater mechanical strength and cytocompatibility than the pure fibroin scaffolds (Baldino et al., 2015). During the formation of SF/HAp, the β-sheets crystal in the SF act as a nucleation site for the deposition of HAp nanocrystals (Vetsch et al., 2015). Bi et al. modified a silk-collagen scaffold with HAp at both ends. The results exhibited massive formation of more mature bone at the tendon-bone interface, more collagen I and osteocalcin deposition, bone mineral formation, and better osteoarthritis prevention in the modified group than the silk-collagen group (Bi et al., 2015). Chop fiber (CF), nanohydroxyapatite (n-HA), and silk fibroin (SF) porous hybrid scaffolds (SHCF) were produced by Jin et al. utilizing freeze-drying. The mechanical properties of composite scaffolds can be improved significantly by doping CF and n-HA. The scaffold can stimulate the growth and osteogenic differentiation of BMSCs by upregulating the expression of Capns1 and controlling calcium signals, which in turn promotes bone repair, as demonstrated by experiments conducted on cells and mice (Jin et al., 2023).

Compared with pure alginate and alginate/HAp, alginate/HAp/SF composites showed significantly higher new bone formation and decreased relative TNF-α levels (Jo et al., 2017). Similarly, in the 3D porous HAp/SF/sodium alginate scaffold, a higher ratio of SF/HAp to sodium alginate resulted in improved cell proliferation and enhanced alkaline phosphatase activity. In another study, the graphene oxide-modified SF/nano-HAp scaffold loaded with urine-derived stem cells could promote bone regeneration and had immunomodulatory effects (Sun et al., 2021). Furthermore, aluminum oxide nanoparticle-containing SF composite scaffolds increased the expression of osteogenic markers in rabbit adipose stem cells (Zafar et al., 2020). Besides HAp, alginate, graphene oxide, and some other substances, such as Ca^+^, Ti and Mg^+^, were added to the SF scaffold for a better bone repair effect (Türkkan et al., 2017; Gao et al., 2018; Johari, Madaah Hosseini and Samadikuchaksaraei, 2020; Pandey et al., 2021; Li et al., 2024).

### 4.3 Cartilage regeneration

The research of SF used in cartilage repair started decades before (Cheng et al., 2018; Ribeiro et al., 2018; Farokhi et al., 2019). Aoki et al. confirmed the proliferation and differentiation phenotype of chondrocytes in the SF sponge (Aoki et al., 2003). Pore size and porosity significantly affected cell attachment and penetration in SF-based scaffolds for cartilage and osteochondral tissue regeneration. The pore size below 300 μm helped endochondral ossification, whereas the size greater than 300 μm increased osteogenesis (Rasheed et al., 2019).

SF scaffolds could act as a release system to promote cartilage differentiation. Wu et al., 2020 designed an Rb1/TGF-β1 loaded SF-gelatin porous scaffold (GSTR). It created a microenvironment for cartilage regeneration to promote chondrogenesis, suppress the inflammation levels *in vivo* and enhance hyaline cartilage regeneration *in vitro*. TGF-β3 was also wrapped in SF scaffolds (S/D) to expedite the chondrogenic differentiation of adipose-derived stem cells *in vitro* (Yang et al., 2017). Li developed SF hydrogel scaffolds incorporated with bone morphogenetic protein-2 (BMP-2). The scaffold enhanced BMSCs’ capacity to produce cartilage both *in vivo* and *in vitro* (Li et al., 2021). Chen and colleagues synthesized an SF scaffold loaded with tanshinone IIA (TAN). The release of TAN can augment the transcription of genes linked to chondrocyte activity in chondrocytes and mitigate oxidative stress, hence fostering cartilage regeneration (Chen et al., 2020).

SF has been blended with other biomaterials to improve the required properties. MSCs seeded on chitosan/SF(CH/SFF) porous scaffold showed significantly higher sulfated glycosaminoglycan deposition and enhanced expression of collagen type Ⅱ and aggrecan in comparison to the pure chitosan scaffold (control) (Singh and Pramanik, 2018). Agrawal et al. discovered that the chondrogenic differentiation of hMSCs progressed more quickly in dynamic culture than in static culture after being seeded on silk-fibroin (SF)/chitosan (CS) scaffolds (Agrawal et al., 2018). Scaffolds with a blend ratio of SF/CS (80:20), pore size in the range 71–210 μm, and a porosity of 82.2% ± 1.3% were found to be superior in supporting cell attachment and viability cell proliferation, and glycosaminoglycan secretion (Vishwanath et al., 2016). Composite scaffolds of filipin protein (SF), gelatin (G), chondroitin sulfate (C), hyaluronic acid (H), and aloe vera (A) were prepared by freeze-drying by Wuttisiriboon et al., 2023 This scaffold has an interconnected porous structure with a pore size of approximately 209 μm. In addition, it has a high absorption rate and good mechanical strength, and can retain its structure for up to 21 days. Cellular experiments also demonstrated that the proliferation of human bone marrow mesenchymal stem cells (BM-MSCs) with this scaffold was significantly higher than that with the SF scaffold. Significantly, Sharafat-Vaziri et al. employed engineered tissue made of autologous chondrocytes and collagen/SF scaffold to do a pilot clinical investigation on two patients with osteochondral lesions in the knee. Clinical evidence has demonstrated the safety and effectiveness of the SF-based scaffold in the healing of large chondral lesions (Sharafat-Vaziri et al., 2020). Although extensive research has been carried out in the laboratory and has demonstrated that SF is a promising material or ingredient for cartilage repair. However, lack of adequate clinical evidence was still the main limitation of its realistic application. Therefore, randomized controlled trials on those materials are required to gather more reliable data about their long-term effects and complications.

### 4.4 Blood vessel tissue regeneration

It is possible to replace or avoid a blocked or damaged blood artery with vascular transplants. Research has confirmed that SF scaffolds supported the growth, adhesion, survival, and expansion of three vascular cells: Human Coronary Artery Endothelial Cells, Human Aortic Smooth Muscle Cells, and Human Aortic Adventitial Fibroblasts (Zhang et al., 2008; Alessandrino and Chiarini, 2019). It was discovered that the benefits of SF-based vascular grafts included their propensity to generate thin luminal layers and their quick reendothelialization (Yamamoto et al., 2016). *In vivo* silk scaffolds have the potential to support blood vessel cell growth, however biological cues are typically required for the cells to penetrate past the surface and into the scaffold’s core (Thurber et al., 2015).

The ability of blood vessels to grow within silk scaffolds varies based on different SF processing and scaffold morphology. Diameter and porosity are common influencing factors for cell behavior, such as cell infiltration, attachment, and proliferation (Ghasemi-Mobarakeh et al., 2015). Sun et al. fabricated SF tubular scaffolds with different pore sizes, the micropores of 30–50 µm were found to be suitable for the growth and proliferation of human umbilical vein endothelial cell (HUVEC) (Sun et al., 2016). Polytetrafluoroethylene (PTFE, Teflon) and ethylene terephthalate (PET, Dacron) grafts performed well for large vessels but had less than 50% patency for small vessel occlusions due to acute thrombosis (Thurber et al., 2015). Compared with traditional PTFE vascular grafts, the SF-based scaffold led to better reendothelialization and higher patency rate (94.7% in the SF group vs. 80.0% in the PTFE group) at 4 weeks after replacing the inferior vena cava of a rat (Kiritani et al., 2020). A vital barrier against thrombosis-confluent endothelium was created by smooth muscle and endothelial cells migrating into and multiplying within the silk grafts (Lovett et al., 2010). Compared to SF vascular grafts (inner diameter 3.5 mm) and ePTFE grafts (inner diameter 4 mm) on the carotid artery in beagles, there was no significant difference in vascular repair effect between the two groups at 3 months, the patency rates of the SF and expanded PTFE grafts were 7.8% and 0% at 6 months, respectively (Haga et al., 2017). SF grafts with a smaller inner diameter (1.5 mm) also had significantly higher patency rates 1 year after implantation than expanded PTFE grafts (85.1% vs. 30%)(Enomoto et al., 2010). Both materials have their advantages: the rapid reendothelialization of expanded PTFE graft reduced intimal blockages, while SF’s hydrophilicity and cytocompatibility improved the proliferation of HUVEC. Yan et al. functionalized the ePTFE graft with SF hydrogel and obtained improved proliferation of HUVEC as SF is cytocompatible and hydrophilic. The complementarity of the two materials obtains better effects (Yan et al., 2021). Tanaka et al. also developed a porous SF coated polyethylene terephthalate (PET) graft with a diameter of less than 6 mm. The results of *in vivo* and *in vitro* experiments demonstrated that the porous SF-coated PET grafts degraded rapidly *in vivo* and facilitated remodeling to their own tissues compared to gelatin-coated PET grafts, making them perfect candidates for commercial grafts (Tanaka et al., 2020). In addition, micro-vessel substitutes (150–300 μm in diameter) made from SF and polyethylene oxide showed cell proliferation and attachment, demonstrating the ability of SF to produce microvascular grafts (Bosio et al., 2017). Apart from the well-known polymers previously described, Yang et al. recently developed a composite scaffold of SF and fibronectin that mimics natural blood vessels, prepared by electrostatic spinning. With a smooth and uniform fiber structure and small fiber diameter, this scaffold exhibited excellent hemocompatibility and an appropriate biodegradation rate, and most importantly, it increased MSC proliferation and adhesion, making it a potentially ideal material for artificial vascular scaffolds (Yang et al., 2024).

Asakura et al. have studied the application of SF materials in vascular repair for many years. The main difference between SF and polyester fibers or expanded PTFE grafts was the unique remodeling function of SF. They coated the knitted SF graft with SF solution and a cross-linking agent poly (ethylene glycol diglycidyl ether), to prepare a small-diameter vascular graft with a diameter of 1.5 mm and a length of 10 mm. The graft exhibited superior physical strength, meanwhile the coating on it can also prevent blood leakage and increase the elasticity (Yagi et al., 2011). In rat abdominal aorta, they later confirmed that the optimum concentration of SF coating was 2.5% (Fukayama et al., 2015). Recently, they conducted *in vivo* experiments with large animal Beagle dogs: SF grafts with a length of 4 cm and inner diameter of 3.5 mm were implanted into the femoral arteries of 6 dogs, and 5 of them showed a high patency rate (Tanaka et al., 2021).

### 4.5 Ligament and tendon regeneration

One important component of the knee joint is the anterior cruciate ligament (ACL). Improper sports and excessive external force can lead to an ACL injury, which causes instability and progressive damage to the knee joint. Artificial ligaments may reduce the danger of disease transfer or morbidity at the donor site associated with autografts or allografts. SF has been confirmed the ability to support adult stem cell differentiation toward ligament lineages (Altman et al., 2002; Bhattacharjee et al., 2017; Bi et al., 2021a; Chen et al., 2021; Sun et al., 2021). SF/collagen composite grafts showed great application potential due to many supportive results. Knitted silk-collagen sponge scaffolds enhanced the expression of ligament matrix genes and promoted collagen fiber assembly, thereby improving the structural and functional repair of ligaments (Chen et al., 2008; Bi et al., 2021b; Saab et al., 2023). Shen et al. investigated the long-term repair effect of silk-collagen scaffolds in a rabbit model of anterior cruciate ligament injury. Migration and adhesion of spindle-shaped cells to the scaffolds were observed 2 months after surgery. After 6 months, a better microstructural morphology was observed. In addition, the knitted silk-collagen sponge scaffold effectively protected the articular surface cartilage and preserved the joint space for 18 months after surgery (Shen et al., 2014). Recently, Geng et al. prepared a SF/collagen three-phase scaffold characterized by a compositional gradient that mimics the natural tendon bone structure, which showed good biocompatibility in cellular experiments while promoting tendon formation (Geng et al., 2024). Another method applied for ligament regeneration was incorporating cells in the SF-based scaffold before implantation to direct ligament bone insertion. The mechanical needs of daily activities could be satisfied by the tensile strength of MSCs and scaffolds (Fan et al., 2008; 2009). Ribeiro et al. presented a biomimetic composite scaffold consisting of horseradish peroxidase crosslinked SF hydrogels, containing ZnSr-doped β-tricalcium phosphate particles. This scaffold possessed sufficient structural integrity, swelling capacity and tensile strength and exhibited cell adhesion, viability and proliferation after 14 days of *in vitro* culture (Ribeiro et al., 2022).

In the clinic, a commercially accessible product for posterior cruciate ligament replacement is the ligament advanced reinforcement system (LARS) composed of polyethylene terephthalate (PET). However, the disadvantages of LARS still exist. For example, it had the complication of arthrofibrosis and heterotopic ossification (Smith et al., 2014; Ranger et al., 2018). Jiang et al., 2016 employed SF to modify the surface of PET to change its hydrophilicity and biocompatibility. A series of *in vitro* experiments confirmed that SF coating enhanced cell adhesion and proliferation and improved the biocompatibility of the material and its process of “ligamentization”. A silk hybrid on the ligamentization was developed for a canine ACL reconstruction in another study. The regenerated ligament showed a compact structure in the silk/PET hybrid group, and there was more regenerated autologous tissue and collagen than PET artificial ligament (Zhi et al., 2019). A stepwise deposition method was used to introduce a multilayer SF coating on the surface of PET artificial ligaments, which was loaded with heparin and bone morphogenetic protein-binding peptide. This triple-coated scaffold not only facilitates the biocompatibility of PET grafts, but also modulates the early inflammatory response in the joint cavity, promotes and improves graft-osteointegration, and shows great potential in improving the clinical efficacy of ACL reconstruction (Chen N. et al., 2023). Silk fibroin could fill up a deficiency in PET to induce the ingrowth of the autologous tissue.

### 4.6 Nerve tissue regeneration

For short gap injuries (<5 mm), the current gold standard in nerve repair surgery was the tension-free end-to-end suture. An autologous nerve graft was a compensatory scheme. However, it was limited by the availability of autologous donor tissue and would lead to lower sensitivity in donor sites, adherent scars, and neuroma formation (Ray and Mackinnon, 2010). Artificial nerve guidance conduits (NGCS) are an alternative strategy for peripheral nerve defects <3.0 cm (Kornfeld et al., 2019).

Many researches were devoted to exploring better nerve defects repair devices by combining SF with other materials such as PLGA (Rao et al., 2017; Namini et al., 2023), polypyrene (PPY)(Sun et al., 2016; Wang et al., 2020), polyethylene oxide (Rajabi et al., 2018) and Collagen (Jiang et al., 2020). Tang et al., 2012 co-cultured dorsal root ganglia and Schwann cells in an SF-based scaffold to form the nerve equivalent of a nerve graft *in vitro*. The graft induced better nerve regeneration and functional recovery than the SF-based scaffold at 12 weeks after nerve grafting. Furthermore, a compelling study evaluated functional and histologic assessments 12 months after implantation of an electrospun SF catheter used to bridge a 30-mm sciatic nerve gap in dogs; the findings demonstrated that SF-based neural scaffolds had acceptable regeneration results, comparable to those of the autografts group (Xue et al., 2018). SilkbridgeTM, a three-layer silk-based 3D tubular architecture for nerve defects repair, was systematically studied and would be the first commercialized silk-based nerve repair product (Alessandrino and Chiarini, 2019; Alessandrino and Fregnan, 2019; Fregnan et al., 2020; Biagiotti et al., 2022; Freddi et al., 2024). The structure of this product is two electrospun layers (inner and outer) and one textile layer (middle), which optimized the mechanical properties and maximized the biological characteristics of the material; it provided a supporting structure to the regenerate axon optimal mechanical properties. Excellent functional and morphological recovery of the median nerve, as well as the absence of inflammation and scar formation, were observed in short-term (2 weeks) and long-term (12 and 24 weeks) animal trials (Fregnan et al., 2020). Before the first-in-human clinical trial, the researchers assessed the mechanical properties, toxicological analysis, and biological safety of Silkbridge. The results confirmed the suitability and biosafety of the device (Bassani et al., 2022). While preclinical trials have yielded positive findings thus far, clinical outcomes are still required to validate safety, effectiveness, and long-term problems in the clinical setting. SF scaffolds have shown promise in promoting nerve regeneration by providing structural support and guidance for axonal growth. Future research may focus on refining scaffold architecture and incorporating neurotrophic factors to enhance nerve regeneration outcomes, particularly in cases of spinal cord injury or peripheral nerve damage.

### 4.7 Other tissue regeneration

In addition, SF has also been explored for some niche tissue repair such as dental, gastrointestinal, urethra, and so on. Xu et al. found that silk scaffolds with 550-micron pore RGD-containing could guide the formation of robust mineralized osteopontin (Xu et al., 2008). However, in their following study, they found that there was no mineralized tissue formation, although silk scaffolds proved to support soft tissue dental pulp formation (Zhang et al., 2011). Pre-seeding cell treatments or sorting and enrichment methods would benefit dental hard tissue regeneration. SF scaffolds loaded with dental pulp stem cells attracted vessels which are crucial for successful healing and regeneration (Woloszyk et al., 2016). Composite scaffolds of SF with PLGA and ketoprofen showed superior anti-inflammatory properties in the treatment of periodontal disease (Chachlioutaki et al., 2022). More interestingly, recent research has shown that SF scaffolds coated with both graphene oxide and reduced graphene oxide can differentiate human dental pulp stem cells by promoting mineralization of the extracellular matrix (Lopez-Garcia et al., 2023).

SF scaffolds are also demonstrated to stimulate small intestinal smooth muscle cells, colon smooth muscle cells, and esophageal smooth muscle cell attachment and contractile differentiation. Hence, the scaffolds were just as effective as tiny intestine submucosa scaffolds at encouraging the adhesion and growth of gastrointestinal epithelial cell lines (Franck et al., 2014). An experiment in a rat model of onlay esophagoplasty suggested that SF scaffolds served as acellular grafts with less inflammation and fibrosis than traditional small intestinal submucosa implants (Algarrahi et al., 2015). However, the result contrasted with an *in vitro* result performed in a porcine defect model for tubular esophagoplasty. In general, bilayer SF graft combined with temporary stenting supported the reformation of tubular neo tissues with contractile and relaxation function. Nonetheless, it appears that BLSF is incompatible with direct tubular esophagoplasty due to the 60% stricture rate and additional problems, such as inadequate innervation and sparse peristaltic function (Gundogdu et al., 2021).

In addition, SF has been applied in urethra repair (Sack et al., 2016). Studies investigated SF scaffold used in a rabbit model for onlay urethroplasty; results showed it promoted smooth muscle and epithelial tissue regeneration with reduced acute inflammation compared with SIS and maintained urethral function for 3 months after implantation (Chung et al., 2014; Algarrahi et al., 2018). Niu et al., 2022 successfully prepared a bionic tubular HA-SF nanofiber scaffold by electrostatic spinning and cross-linking processes, whose structure, morphology, and mechanical properties were close to those of the natural rabbit urethral tissue. The nanofiber surface of this scaffold is more suitable for the growth of UC to form new urinary tract epithelial tissue.

## 5 Conclusion and future outlook

SF scaffolds are widely used in tissue engineering by scientists due to their biocompatibility, biodegradability, mechanical strength, and structural tunability. Scientists have investigated the use of various techniques to fabricate different types of SF scaffold, including thin films, porous scaffolds, and hydrogels, to achieve the desired properties for clinical applications (as shown in Table 1). Researchers are continuously exploring ways to modify silk fibroin to enhance its properties for specific tissue engineering applications. This included surface modifications, blending with other polymers or materials, and functionalization with bioactive molecules to improve cell adhesion, proliferation, and differentiation. The advent of 3D printing technologies has enabled precise control over the architecture and geometry of tissue scaffolds. Although a large number of research articles have been published on the application of SF scaffolds in tissue engineering, however, only a few have reached clinical trials. The U.S. Food and Drug Administration (FDA) has now approved 91 medical devices made from *B. mori* silk. Very few of them are related to SF, and most of the silk-based medical devices are designed for surgical sutures. There are only 7 clinical trials on SF, 4 of which are for wound healing and skin aging applications, and only 2 clinical trials have been completed. There are still issues that need to be addressed before SF scaffolds can be used in clinical trials and commercialized for tissue engineering. Regulatory approvals and expanded manufacturing processes would be important considerations in bringing SF scaffold-based tissue engineering products to market. On one hand, it is difficult to standardize raw materials and their processing procedures. In fact, sericulture is primarily a manual operation. The seasonality and origin of mulberry leaves could have an impact on the batch nature of SF. It seems challenging to manipulate the properties of silk fibroin by controlling these complex variables. On the other hand, new technologies still need to be developed to solve the storage problem in preparation for future commercialization and mass marketing, as SF is difficult to stabilize for long-term preservation. Moreover, the limitations of clinical trials have similarly restricted SF scaffolds. Most of the current clinical trials are confined to skin tissue engineering, while other aspects such as bone tissue are rarely addressed. Meanwhile, very few trials have been conducted for short-and long-term results in large animals (e.g., dogs) and humans, and the current trials have mainly focused on small animals (e.g., rats), which are more compelling and direct evidence. The scientists still need to explore new technologies to transition more SF scaffolds from the laboratory to the clinic in the future. Also, scientists could further focus in the future on generating multifunctional SF scaffolds capable of integrating multiple tissue types or functions within a single scaffold to engineer complex tissues or organs with hierarchical structures and diverse physiological functions. Overall, the future of SF scaffolds in tissue engineering is promising, and investigations are underway to leverage their unique properties to meet a wide range of clinical needs and to advance regenerative medicine towards personalized, functional tissue substitutes.

**TABLE 1:** List of *in vitro*/*in vivo* assessments for silk fibroin-based scaffolds.

Type of engineered tissue	Scaffold composition	Cells used in the *in vitro* studies		Animal models used in the *in vivo* studies	References
Skin	SF, SF/growth factor, SF/stem cells, collagen/SF, SF/PCL/HAM, SF/PU, SF/PLCL, SF/ZO, SF/HA/ZO	Fibroblasts, Endothelial cells, adipose tissue- derived mesenchymal stem cells		Rats, albino mice	Babu et al. (2018), Wöltje et al. (2018), Li et al. (2019), Cui et al. (2020), Hadisi et al. (2020)
Bone	SF/PDLLA/collagen, SF/HAp,SF/HAp/alginate, SF/HAp/graphene oxide	Sem cells		Mouse	Maraldi et al. (2011), Riccio et al. (2012)
Cartilage	SF, SF/gelatin, SF/chitosan	Chondrocytes, Stem cells		Rats	Vishwanath et al. (2016), Yang et al. (2017), Agrawal et al. (2018), Singh and Pramanik (2018), Wu et al. (2020), Li et al. (2021)
Blood Vessel	SF, ePTEE/SF	Human coronary artery endothelial		Rats, Beagle dogs	Zhang et al. (2008), Sun et al. (2016a), Alessandrino and Chiarini (2019), Kiritani et al. (2020), Tanaka et al. (2021)
		cells, aortic	Human smooth		
		muscle cells, and Human aortic adventitial fibroblasts, Human umbilical vein endothelial cell			
Ligament/tendon	SF, SF/collagen, PET/SF	Stem cells		Rabbit	Shen et al. (2014), Zhi et al. (2019)
Nerve	SF/PLGA, SF/PPY, SF/Polyethylene oxide, SF/collagen	Schwann cells		Dogs	Rao et al. (2017), Xue et al. (2018), Jiang et al. (2020)

## References

[B1] Agostinacchio F. Mu X. Dirè S. Motta A. Kaplan D. L. (2021). *In situ* 3D printing: opportunities with silk inks. Trends Biotechnol. 39 (7), 719–730. 10.1016/j.tibtech.2020.11.003 33279280 PMC8169713

[B2] Agrawal P. Pramanik K. Biswas A. Ku Patra R. (2018). *In vitro* cartilage construct generation from silk fibroin-chitosan porous scaffold and umbilical cord blood derived human mesenchymal stem cells in dynamic culture condition. J. Biomed. Mater. Res. Part A 106 (2), 397–407. 10.1002/jbm.a.36253 28960800

[B3] Ahsan F. Ansari T. Usmani S. Bagga P. (2018). An insight on silk protein sericin: from processing to biomedical application. Drug Res. 68 (06), 317–327. 10.1055/s-0043-121464 29132177

[B4] Alessandrino A. Chiarini A. Biagiotti M. Dal Prà I. Bassani G. A. Vincoli V. (2019). Three-layered silk fibroin tubular scaffold for the repair and regeneration of small caliber blood vessels: from design to *in vivo* pilot tests. Front. Bioeng. Biotechnol. 7, 356. 10.3389/fbioe.2019.00356 31850325 PMC6895545

[B5] Alessandrino A. Fregnan F. Biagiotti M. Muratori L. Bassani G. A. Ronchi G. (2019). SilkBridge^TM^: a novel biomimetic and biocompatible silk-based nerve conduit. Biomaterials Sci. 7 (10), 4112–4130. 10.1039/c9bm00783k 31359013

[B6] Algarrahi K. Affas S. Sack B. S. Yang X. Costa K. Seager C. (2018). Repair of injured urethras with silk fibroin scaffolds in a rabbit model of onlay urethroplasty. J. Surg. Res. 229, 192–199. 10.1016/j.jss.2018.04.006 29936989 PMC6022288

[B7] Algarrahi K. Franck D. Ghezzi C. E. Cristofaro V. Yang X. Sullivan M. P. (2015). Acellular bi-layer silk fibroin scaffolds support functional tissue regeneration in a rat model of onlay esophagoplasty. Biomaterials 53, 149–159. 10.1016/j.biomaterials.2015.02.092 25890715 PMC4405663

[B8] Altman G. H. Diaz F. Jakuba C. Calabro T. Horan R. L. Chen J. (2003). Silk-based biomaterials. Biomaterials 24 (3), 401–416. 10.1016/s0142-9612(02)00353-8 12423595

[B9] Altman G. H. Horan R. L. Lu H. H. Moreau J. Martin I. Richmond J. C. (2002). Silk matrix for tissue engineered anterior cruciate ligaments. Biomaterials 23 (20), 4131–4141. 10.1016/s0142-9612(02)00156-4 12182315

[B10] Anand P. Pandey J. P. Pandey D. M. (2021). Study on cocoonase, sericin, and degumming of silk cocoon: computational and experimental. J. Genet. Eng. Biotechnol. 19 (1), 32. 10.1186/s43141-021-00125-2 33594479 PMC7886927

[B11] Aoki H. Tomita N. Morita Y. Hattori K. Harada Y. Sonobe M. (2003). Culture of chondrocytes in fibroin--hydrogel sponge. Bio-Medical Mater. Eng. 13 (4), 309–316. 14646046

[B12] Baba A. Matsushita S. Kitayama K. Asakura T. Sezutsu H. Tanimoto A. (2019). Silk fibroin produced by transgenic silkworms overexpressing the Arg-Gly-Asp motif accelerates cutaneous wound healing in mice. J. Biomed. Mater. Res. Part B Appl. Biomaterials 107 (1), 97–103. 10.1002/jbm.b.34098 29504231

[B13] Babu P. J. Doble M. Raichur A. M. (2018). Silver oxide nanoparticles embedded silk fibroin spuns: microwave mediated preparation, characterization and their synergistic wound healing and anti-bacterial activity. J. colloid interface Sci. 513, 62–71. 10.1016/j.jcis.2017.11.001 29132106

[B14] Baldino L. Naddeo F. Cardea S. Naddeo A. Reverchon E. (2015). FEM modeling of the reinforcement mechanism of hydroxyapatite in PLLA scaffolds produced by supercritical drying, for tissue engineering applications. J. Mech. Behav. Biomed. Mater. 51, 225–236. 10.1016/j.jmbbm.2015.07.021 26275485

[B15] Bassani G. A. Vincoli V. Biagiotti M. Valsecchi E. Zucca M. V. Clavelli C. (2022). A route to translate a silk-based medical device from lab to clinic: the silk biomaterials srl experience. Insects 13 (2), 212. 10.3390/insects13020212 35206785 PMC8875467

[B16] Bazrafshan A. Owji M. Yazdani M. Varedi M. (2014). Activation of mitosis and angiogenesis in diabetes-impaired wound healing by processed human amniotic fluid. J. Surg. Res. 188 (2), 545–552. 10.1016/j.jss.2014.01.041 24582064

[B17] Belbéoch C. Lejeune J. Vroman P. Salaün F. (2021). Silkworm and spider silk electrospinning: a review. Environ. Chem. Lett. 19, 1737–1763. 10.1007/s10311-020-01147-x 33424525 PMC7779161

[B18] Berthiaume F. Maguire T. J. Yarmush M. L. (2011). Tissue engineering and regenerative medicine: history, progress, and challenges. Annu. Rev. Chem. Biomol. Eng. 2, 403–430. 10.1146/annurev-chembioeng-061010-114257 22432625

[B19] Bhardwaj N. Kundu S. C. (2010). Electrospinning: a fascinating fiber fabrication technique. Biotechnol. Adv. 28 (3), 325–347. 10.1016/j.biotechadv.2010.01.004 20100560

[B20] Bhardwaj N. Sow W. T. Devi D. Ng K. W. Mandal B. B. Cho N. J. (2015). Silk fibroin--keratin based 3D scaffolds as a dermal substitute for skin tissue engineering. Integr. Biol. 7 (1), 53–63. 10.1039/c4ib00208c 25372050

[B21] Bhattacharjee P. Kundu B. Naskar D. Kim H. W. Maiti T. K. Bhattacharya D. (2017). Silk scaffolds in bone tissue engineering: an overview. Acta biomater. 63, 1–17. 10.1016/j.actbio.2017.09.027 28941652

[B22] Bi F. Chen Y. Liu J. Hu W. Tian K. (2021b). Bone mesenchymal stem cells contribute to ligament regeneration and graft--bone healing after anterior cruciate ligament reconstruction with silk--collagen scaffold. Stem Cells Int. 2021, 1–11. 10.1155/2021/6697969 PMC8088362 33981343

[B23] Bi F. Chen Y. Liu J. Wang Y. Xu D. Tian K. (2021a). Anterior cruciate ligament reconstruction in a rabbit model using a silk-collagen scaffold modified by hydroxyapatite at both ends: a histological and biomechanical study. J. Orthop. Surg. Res. 16, 139–212. 10.1186/s13018-021-02281-0 33593365 PMC7885370

[B24] Bi F. Shi Z. Liu A. Guo P. Yan S. (2015). Anterior cruciate ligament reconstruction in a rabbit model using silk-collagen scaffold and comparison with autograft. PloS one 10 (5), e0125900. 10.1371/journal.pone.0125900 25938408 PMC4418759

[B25] Biagiotti M. Bassani G. A. Chiarini A. Vincoli V. T. Dal Prà I. Cosentino C. (2022). Electrospun silk fibroin scaffolds for tissue regeneration: chemical, structural, and toxicological implications of the formic acid-silk fibroin interaction. Front. Bioeng. Biotechnol. 10, 833157. 10.3389/fbioe.2022.833157 35155396 PMC8829063

[B26] Bosio V. E. Brown J. Rodriguez M. J. Kaplan D. L. (2017). Biodegradable porous silk microtubes for tissue vascularization. J. Mater. Chem. B 5 (6), 1227–1235. 10.1039/c6tb02712a 28944059 PMC5604870

[B27] Chachlioutaki K. Karavasili C. Adamoudi E. Bouropoulos N. Tzetzis D. Bakopoulou A. (2022). Silk sericin/PLGA electrospun scaffolds with anti-inflammatory drug-eluting properties for periodontal tissue engineering. Biomater. Adv. 133, 112723. 10.1016/j.msec.2022.112723 35474147

[B28] Chen J. Mo Q. Sheng R. Zhu A. Ling C. Luo Y. (2021a). The application of human periodontal ligament stem cells and biomimetic silk scaffold for *in situ* tendon regeneration. Stem Cell Res. \& Ther. 12, 596–615. 10.1186/s13287-021-02661-7 34863301 PMC8642874

[B29] Chen K. Li Y. Li Y. Pan W. Tan G. (2023a). Silk fibroin combined with electrospinning as a promising strategy for tissue regeneration. Macromol. Biosci. 23 (2), 2200380. 10.1002/mabi.202200380 36409150

[B30] Chen N. Jin W. Gao H. Hong J. Sun L. Yao J. (2023b). Sequential intervention of anti-inflammatory and osteogenesis with silk fibroin coated polyethylene terephthalate artificial ligaments for anterior cruciate ligament reconstruction. J. Mater. Chem. B 11 (34), 8281–8290. 10.1039/d3tb00911d 37584321

[B31] Chen W. Xu Y. Li H. Dai Y. Zhou G. Zhou Z. (2020). Tanshinone IIA delivery silk fibroin scaffolds significantly enhance articular cartilage defect repairing via promoting cartilage regeneration. ACS Appl. Mater. \& interfaces 12 (19), 21470–21480. 10.1021/acsami.0c03822 32314911

[B32] Chen X. Qi Y. Y. Wang L. L. Yin Z. Yin G. L. Zou X. H. (2008). Ligament regeneration using a knitted silk scaffold combined with collagen matrix. Biomaterials 29 (27), 3683–3692. 10.1016/j.biomaterials.2008.05.017 18541295

[B33] Chen Y. Xu W. Shafiq M. Tang J. Hao J. Xie X. (2021b). Three-dimensional porous gas-foamed electrospun nanofiber scaffold for cartilage regeneration. J. Colloid Interface Sci. 603, 94–109. 10.1016/j.jcis.2021.06.067 34197994

[B34] Cheng G. Davoudi Z. Xing X. Yu X. Cheng X. Li Z. (2018). Advanced silk fibroin biomaterials for cartilage regeneration. ACS Biomaterials Sci. \& Eng. 4 (8), 2704–2715. 10.1021/acsbiomaterials.8b00150 33434996

[B35] Chimene D. Lennox K. K. Kaunas R. R. Gaharwar A. K. (2016). Advanced bioinks for 3D printing: a materials science perspective. Ann. Biomed. Eng. 44, 2090–2102. 10.1007/s10439-016-1638-y 27184494

[B36] Chizari M. Khosravimelal S. Tebyaniyan H. Moosazadeh Moghaddam M. Gholipourmalekabadi M. (2022). Fabrication of an antimicrobial peptide-loaded silk fibroin/gelatin bilayer sponge to apply as a wound dressing; an *in vitro* study. Int. J. Peptide Res. Ther. 28, 18–13. 10.1007/s10989-021-10333-6

[B37] Choi J. H. Kim D. K. Song J. E. Oliveira J. M. Reis R. L. Khang G. (2018). Silk fibroin-based scaffold for bone tissue engineering. Nov. Biomaterials Regen. Med. 1077, 371–387. 10.1007/978-981-13-0947-2_20 30357699

[B38] Chung Y. G. Tu D. Franck D. Gil E. S. Algarrahi K. Adam R. M. (2014). Acellular bi-layer silk fibroin scaffolds support tissue regeneration in a rabbit model of onlay urethroplasty. PloS one 9 (3), e91592. 10.1371/journal.pone.0091592 24632740 PMC3954771

[B39] Compaan A. M. Christensen K. Huang Y. (2017). Inkjet bioprinting of 3D silk fibroin cellular constructs using sacrificial alginate. ACS Biomaterials Sci. \& Eng. 3 (8), 1519–1526. 10.1021/acsbiomaterials.6b00432 33429638

[B40] Costantini M. Barbetta A. (2018). Gas foaming technologies for 3D scaffold engineering. Functional 3D tissue engineering scaffolds. Elsevier. 127–149.

[B41] Craig C. L. (1997). Evolution of arthropod silks. Annu. Rev. entomology 42 (1), 231–267. 10.1146/annurev.ento.42.1.231 15012314

[B42] Cui B. Zhang C. Gan B. Liu W. Liang J. Fan Z. (2020). Collagen-tussah silk fibroin hybrid scaffolds loaded with bone mesenchymal stem cells promote skin wound repair in rats. Mater. Sci. Eng. C 109, 110611. 10.1016/j.msec.2019.110611 32228999

[B43] Das S. Pati F. Choi Y. J. Rijal G. Shim J. H. Kim S. W. (2015). Bioprintable, cell-laden silk fibroin--gelatin hydrogel supporting multilineage differentiation of stem cells for fabrication of three-dimensional tissue constructs. Acta biomater. 11, 233–246. 10.1016/j.actbio.2014.09.023 25242654

[B44] DeBari M. K. King C. I. Altgold T. A. Abbott R. D. (2021). Silk fibroin as a green material. ACS Biomaterials Sci. \& Eng. 7 (8), 3530–3544. 10.1021/acsbiomaterials.1c00493 34260194

[B45] Dehghani N. Haghiralsadat F. Yazdian F. Sadeghian-Nodoushan F. Ghasemi N. Mazaheri F. (2023). Chitosan/silk fibroin/nitrogen-doped carbon quantum dot/α-tricalcium phosphate nanocomposite electrospinned as a scaffold for wound healing application: *in vitro* and *in vivo* studies. Int. J. Biol. Macromol. 238, 124078. 10.1016/j.ijbiomac.2023.124078 36944378

[B46] Dong M. Mao Y. Zhao Z. Zhang J. Zhu L. Chen L. (2022). Novel fabrication of antibiotic containing multifunctional silk fibroin injectable hydrogel dressing to enhance bactericidal action and wound healing efficiency on burn wound: *in vitro* and *in vivo* evaluations. Int. Wound J. 19 (3), 679–691. 10.1111/iwj.13665 34414663 PMC8874045

[B47] Dong T. Mi R. Wu M. Zhong N. Zhao X. Chen X. (2019). The regenerated silk fibroin hydrogel with designed architecture bioprinted by its microhydrogel. J. Mater. Chem. B 7 (27), 4328–4337. 10.1039/c9tb00783k

[B48] Enomoto S. Sumi M. Kajimoto K. Nakazawa Y. Takahashi R. Takabayashi C. (2010). Long-term patency of small-diameter vascular graft made from fibroin, a silk-based biodegradable material. J. Vasc. Surg. 51 (1), 155–164. 10.1016/j.jvs.2009.09.005 19954921

[B49] Fan H. Liu H. Toh S. L. Goh J. C. (2009). Anterior cruciate ligament regeneration using mesenchymal stem cells and silk scaffold in large animal model. Biomaterials 30 (28), 4967–4977. 10.1016/j.biomaterials.2009.05.048 19539988

[B50] Fan H. Liu H. Wong E. J. Toh S. L. Goh J. C. (2008). *In vivo* study of anterior cruciate ligament regeneration using mesenchymal stem cells and silk scaffold. Biomaterials 29 (23), 3324–3337. 10.1016/j.biomaterials.2008.04.012 18462787

[B51] Farokhi M. Mottaghitalab F. Fatahi Y. Saeb M. R. Zarrintaj P. Kundu S. C. (2019). Silk fibroin scaffolds for common cartilage injuries: possibilities for future clinical applications. Eur. Polym. J. 115, 251–267. 10.1016/j.eurpolymj.2019.03.035

[B52] Franck D. Chung Y. G. Coburn J. Kaplan D. L. Estrada C. R. Mauney J. R. (2014). *In vitro* evaluation of bi-layer silk fibroin scaffolds for gastrointestinal tissue engineering. J. tissue Eng. 5, 204173141455684. 10.1177/2041731414556849 PMC4228923 25396043

[B53] Freddi G. Pisani V. Francavilla G. Alessandrino A. (2024). Translation of a silk-based medical device from bench to bedside. Silk-Based Biomaterials Tissue Eng. Regen. Precis. Med., 805–832. 10.1016/b978-0-323-96017-5.00023-6

[B54] Fregnan F. Muratori L. Bassani G. A. Crosio A. Biagiotti M. Vincoli V. (2020). Preclinical validation of SilkBridgeTM for peripheral nerve regeneration. Front. Bioeng. Biotechnol. 8, 835. 10.3389/fbioe.2020.00835 32850714 PMC7426473

[B55] Fukayama T. Ozai Y. Shimokawadoko H. Aytemiz D. Tanaka R. Machida N. (2015). Effect of fibroin sponge coating on *in vivo* performance of knitted silk small diameter vascular grafts. Organogenesis 11 (3), 137–151. 10.1080/15476278.2015.1093268 26496652 PMC4879894

[B56] Gai T. Tong X. Han M. Li C. Fang C. Zou Y. (2020). Cocoonase is indispensable for Lepidoptera insects breaking the sealed cocoon. PLoS Genet. 16 (9), e1009004. 10.1371/journal.pgen.1009004 32986696 PMC7544147

[B57] Gaidhani K. A. Deepak B. Harwalker M. Nirgude P. S. Zhou Y. Qi K. (2015). Lyophilization/freeze drying--a review. World J. Pharm. Res. 4 (8), 516–543.

[B58] Gao Y. Shao W. Qian W. He J. Zhou Y. Qi K. (2018). Biomineralized poly (l-lactic-co-glycolic acid)-tussah silk fibroin nanofiber fabric with hierarchical architecture as a scaffold for bone tissue engineering. Mater. Sci. Eng. C 84, 195–207. 10.1016/j.msec.2017.11.047 29519429

[B59] Garg K. Bowlin G. L. (2011). Electrospinning jets and nanofibrous structures. Biomicrofluidics 5 (1), 13403. 10.1063/1.3567097 21522493 PMC3082340

[B60] Geng Y. Cui P. Hu M. Zhang B. Dai L. Han F. (2024). Biomimetic triphasic silk fibroin scaffolds seeded with tendon-derived stem cells for tendon-bone junction regeneration. Biomaterials Sci. 12, 1239–1248. [Preprint]. 10.1039/d3bm00548h 38231128

[B61] Ghanbari E. Mehdipour A. Khazaei M. Khoshfeterat A. b. Niknafs B. (2023). A review of recent advances on osteogenic applications of Silk fibroin as a potential bio-scaffold in bone tissue engineering. Int. J. Polym. Mater. Polym. Biomaterials 72 (9), 665–680. 10.1080/00914037.2022.2032707

[B62] Ghasemi-Mobarakeh L. Prabhakaran M. P. Tian L. Elham S.-J. Leila D. Seeram R. (2015). Structural properties of scaffolds: crucial parameters towards stem cells differentiation. World J. stem cells 7 (4), 728. 10.4252/wjsc.v7.i4.728 26029344 PMC4444613

[B63] Grinberg O. Binderman I. Bahar H. Zilberman M. (2010). Highly porous bioresorbable scaffolds with controlled release of bioactive agents for tissue-regeneration applications. Acta biomater. 6 (4), 1278–1287. 10.1016/j.actbio.2009.10.047 19887123

[B64] Gundogdu G. Morhardt D. Cristofaro V. Algarrahi K. Yang X. Costa K. (2021). Evaluation of bilayer silk fibroin grafts for tubular esophagoplasty in a porcine defect model. Tissue Eng. Part A 27 (1–2), 103–116. 10.1089/ten.tea.2020.0061 32460641 PMC7826443

[B65] Gupta P. Mandal B. B. (2021). Silk biomaterials for vascular tissue engineering applications. Acta biomater. 134, 79–106. 10.1016/j.actbio.2021.08.004 34384912

[B66] Hadisi Z. Farokhi M. Bakhsheshi‐Rad H. R. Jahanshahi M. Hasanpour S. Pagan E. (2020). Hyaluronic acid (HA)-based silk fibroin/zinc oxide core--shell electrospun dressing for burn wound management. Macromol. Biosci. 20 (4), 1900328. 10.1002/mabi.201900328 32077252

[B67] Haga M. Yamamoto S. Okamoto H. Hoshina K. Asakura T. Watanabe T. (2017). Histological reactions and the *in vivo* patency rates of small silk vascular grafts in a canine model. Ann. Vasc. Dis. 10 (2), 132–138. 10.3400/avd.oa.16-00118 29034039 PMC5579779

[B68] Hajiabbas M. Alemzadeh I. Vossoughi M. (2020). A porous hydrogel-electrospun composite scaffold made of oxidized alginate/gelatin/silk fibroin for tissue engineering application. Carbohydr. Polym. 245, 116465. 10.1016/j.carbpol.2020.116465 32718603

[B69] Hashimoto T. Kojima K. Tamada Y. (2020). Higher gene expression related to wound healing by fibroblasts on silk fibroin biomaterial than on collagen. Molecules 25 (8), 1939. 10.3390/molecules25081939 32331316 PMC7221890

[B70] He S.-J. Valluzzi R. Gido S. P. (1999). Silk I structure in *Bombyx mori* silk foams. Int. J. Biol. Macromol. 24 (2–3), 187–195. 10.1016/s0141-8130(99)00004-5 10342764

[B71] Hodgkinson T. D. (2014). Silk fibroin biomaterials for skin tissue engineering applications. United Kingdom: The University of Manchester.

[B72] Hölzl K. Lin S. Tytgat L. Van Vlierberghe S. Gu L. Ovsianikov A. (2016). Bioink properties before, during and after 3D bioprinting. Biofabrication 8 (3), 032002. 10.1088/1758-5090/8/3/032002 27658612

[B73] Huang K. Jinzhong Z. Zhu T. Morsi Y. Aldalbahi A. (2020). PLCL/Silk fibroin based antibacterial nano wound dressing encapsulating oregano essential oil: fabrication, characterization and biological evaluation. Colloids Surfaces B Biointerfaces 196, 111352. 10.1016/j.colsurfb.2020.111352 32919244

[B74] Huang T.-Y. Wang G. S. Tseng C. C. Su W. T. (2019). Epidermal cells differentiated from stem cells from human exfoliated deciduous teeth and seeded onto polyvinyl alcohol/silk fibroin nanofiber dressings accelerate wound repair. Mater. Sci. Eng. C 104, 109986. 10.1016/j.msec.2019.109986 31499995

[B75] Humenik M. Lang G. Scheibel T. (2018). Silk nanofibril self-assembly versus electrospinning. Wiley Interdiscip. Rev. Nanomedicine Nanobiotechnology 10 (4), e1509. 10.1002/wnan.1509 29393590

[B76] Jabbari F. Hesaraki S. Houshmand B. (2019). The physical, mechanical, and biological properties of silk fibroin/chitosan/reduced graphene oxide composite membranes for guided bone regeneration. J. Biomaterials Sci. Polym. Ed. 30 (18), 1779–1802. 10.1080/09205063.2019.1666235 31506050

[B77] Janik H. Marzec M. (2015). A review: fabrication of porous polyurethane scaffolds. Mater. Sci. Eng. C 48, 586–591. 10.1016/j.msec.2014.12.037 25579961

[B78] Jiang J. Ai C. Zhan Z. Zhang P. Wan F. Chen J. (2016). Enhanced fibroblast cellular ligamentization process to polyethylene terepthalate artificial ligament by silk fibroin coating. Artif. organs 40 (4), 385–393. 10.1111/aor.12571 26526301

[B79] Jiang J. Carlson M. A. Teusink M. J. Wang H. MacEwan M. R. Xie J. (2015). Expanding two-dimensional electrospun nanofiber membranes in the third dimension by a modified gas-foaming technique. ACS Biomaterials Sci. \& Eng. 1 (10), 991–1001. 10.1021/acsbiomaterials.5b00238 PMC13439639 33429530

[B80] Jiang J.-P. Zhang S. Liu X. Y. Zhao F. Zhu X. (2020). Three-dimensional bioprinting collagen/silk fibroin scaffold combined with neural stem cells promotes nerve regeneration after spinal cord injury. Neural Regen. Res. 15 (5), 959–968. 10.4103/1673-5374.268974 31719263 PMC6990792

[B81] Jin S. Fu X. Zeng W. Chen A. Luo Z. Li Y. (2023). Chopped fibers and nano-hydroxyapatite enhanced silk fibroin porous hybrid scaffolds for bone augmentation. J. Mater. Chem. B 11 (7), 1557–1567. 10.1039/d2tb02510h 36692356

[B82] Jo Y.-Y. Kim S. G. Kwon K. J. Kweon H. Chae W. S. Yang W. G. (2017). Silk fibroin-alginate-hydroxyapatite composite particles in bone tissue engineering applications *in vivo* . Int. J. Mol. Sci. 18 (4), 858. 10.3390/ijms18040858 28420224 PMC5412440

[B83] Johari N. Madaah Hosseini H. R. Samadikuchaksaraei A. (2020). Mechanical modeling of silk fibroin/TiO2 and silk fibroin/fluoridated TiO2 nanocomposite scaffolds for bone tissue engineering. Iran. Polym. J. 29 (3), 219–224. 10.1007/s13726-020-00789-6

[B84] Kim H. J. Kim M. K. Lee K. H. Nho S. K. Han M. S. Um I. C. (2017). Effect of degumming methods on structural characteristics and properties of regenerated silk. Int. J. Biol. Macromol. 104, 294–302. 10.1016/j.ijbiomac.2017.06.019 28601646

[B85] Kim S. H. Yeon Y. K. Lee J. M. Chao J. R. Lee Y. J. Seo Y. B. (2018). Precisely printable and biocompatible silk fibroin bioink for digital light processing 3D printing. Nat. Commun. 9 (1), 1620. 10.1038/s41467-018-03759-y 29693652 PMC5915392

[B86] Kiritani S. Kaneko J. Ito D. Morito M. Ishizawa T. Akamatsu N. (2020). Silk fibroin vascular graft: a promising tissue-engineered scaffold material for abdominal venous system replacement. Sci. Rep. 10 (1), 21041. 10.1038/s41598-020-78020-y 33273511 PMC7713399

[B87] Kornfeld T. Vogt P. M. Radtke C. (2019). Nerve grafting for peripheral nerve injuries with extended defect sizes. Wien. Med. Wochenschr. (1946) 169 (9), 240–251. 10.1007/s10354-018-0675-6 PMC6538587 30547373

[B88] Kramschuster A. Turng L.-S. (2012). 17—fabrication of tissue engineering scaffolds. Handb. Biopolymers Biodegrad. Plastics Prop. Process. Appl. 427.

[B89] Kuboyama N. Kiba H. Arai K. Uchida R. Tanimoto Y. Bhawal U. K. (2013). Silk fibroin-based scaffolds for bone regeneration. J. Biomed. Mater. Res. Part B Appl. Biomaterials 101 (2), 295–302. 10.1002/jbm.b.32839 23125151

[B90] Kundu B. Rajkhowa R. Kundu S. C. Wang X. (2013). Silk fibroin biomaterials for tissue regenerations. Adv. drug Deliv. Rev. 65 (4), 457–470. 10.1016/j.addr.2012.09.043 23137786

[B91] Kunz R. I. Brancalhão R. M. C. Ribeiro L. d. F. C. Natali M. R. M. (2016). Silkworm sericin: properties and biomedical applications. BioMed Res. Int. 2016, 1–19. 10.1155/2016/8175701 PMC5124675 27965981

[B92] Lee H. Yang G. H. Kim M. Lee J. Huh J. Kim G. (2018). Fabrication of micro/nanoporous collagen/dECM/silk-fibroin biocomposite scaffolds using a low temperature 3D printing process for bone tissue regeneration. Mater. Sci. Eng. C 84, 140–147. 10.1016/j.msec.2017.11.013 29519423

[B93] Lehmann T. Vaughn A. E. Seal S. Liechty K. W. Zgheib C. (2022). Silk fibroin-based therapeutics for impaired wound healing. Pharmaceutics 14 (3), 651. 10.3390/pharmaceutics14030651 35336024 PMC8949428

[B94] Li J. Zhang S. He C. Ling J. (2024a). Electrospun fibers based anisotropic silk fibroin film with photodynamic antibacterial therapy for *S. aureus* infected wound healing. Int. J. Biol. Macromol. 254, 127685. 10.1016/j.ijbiomac.2023.127685 38287584

[B95] Li M. You J. Qin Q. Liu M. Yang Y. Jia K. (2023). A comprehensive review on silk fibroin as a persuasive biomaterial for bone tissue engineering. Int. J. Mol. Sci. 24 (3), 2660. 10.3390/ijms24032660 36768980 PMC9917095

[B96] Li M. Zhong L. He W. Ding Z. Hou Q. Zhao Y. (2019). Concentrated conditioned medium-loaded silk nanofiber hydrogels with sustained release of bioactive factors to improve skin regeneration. ACS Appl. Bio Mater. 2 (10), 4397–4407. 10.1021/acsabm.9b00611 35021399

[B97] Li X. Hajinur Hirad A. Alarfaj A. A. Li H. Santhanam R. (2024b). A convergent fabrication of graphene oxide/silk fibroin/hydroxyapatite nanocomposites delivery improved early osteoblast cell adhesion and bone regeneration. Arabian J. Chem. 17 (2), 105468. 10.1016/j.arabjc.2023.105468

[B98] Li X. You R. Luo Z. Chen G. Li M. (2016). Silk fibroin scaffolds with a micro-/nano-fibrous architecture for dermal regeneration. J. Mater. Chem. B 4 (17), 2903–2912. 10.1039/c6tb00213g 32262968

[B99] Li Y. Liu Y. Guo Q. (2021). Silk fibroin hydrogel scaffolds incorporated with chitosan nanoparticles repair articular cartilage defects by regulating TGF-β1 and BMP-2. Arthritis Res. \& Ther. 23 (1), 50. 10.1186/s13075-020-02382-x 33531052 PMC7856775

[B100] Liu L. Zhang S. Huang J. (2019). Progress in modification of silk fibroin fiber. Sci. China Technol. Sci. 62, 919–930. 10.1007/s11431-018-9508-3

[B101] Liu X. Huang Q. Pan P. Fang M. Zhang Y. Yang S. (2023). Comparative study of the preparation of high-molecular-weight fibroin by degumming silk with several neutral proteases. Polymers 15 (16), 3383. 10.3390/polym15163383 37631440 PMC10459046

[B102] Lopez-Garcia S. Aznar-Cervantes S. D. Pagán A. Llena C. Forner L. Sanz J. L. (2023). 3D Graphene/silk fibroin scaffolds enhance dental pulp stem cell osteo/odontogenic differentiation. Dent. Mater. 40, 431–440. [Preprint]. 10.1016/j.dental.2023.12.009 38114344

[B103] Lovett M. Eng G. Kluge J. Cannizzaro C. Vunjak-Novakovic G. Kaplan D. L. (2010). Tubular silk scaffolds for small diameter vascular grafts. Organogenesis 6 (4), 217–224. 10.4161/org.6.4.13407 21220960 PMC3055647

[B104] Lozano-Pérez A. A. Montalbán M. G. Aznar‐Cervantes S. D. Cragnolini F. Cenis J. L. Víllora G. (2015). Production of silk fibroin nanoparticles using ionic liquids and high-power ultrasounds. J. Appl. Polym. Sci. 132 (12). 10.1002/app.41702

[B105] Ma L. (2003). Collagen/chitosan porous scaffolds with improved biostability for skin tissue engineering. Biomaterials 24 (26), 4833–4841. 10.1016/s0142-9612(03)00374-0 14530080

[B106] Maniglio D. Bonani W. Migliaresi C. Motta A. (2018). Silk fibroin porous scaffolds by N2O foaming. J. Biomaterials Sci. 29 (5), 491–506. *Polymer edition* . 10.1080/09205063.2018.1423811 29297760

[B107] Maraldi T. Riccio M. Resca E. Pisciotta A. La Sala G. B. Ferrari A. (2011). Human amniotic fluid stem cells seeded in fibroin scaffold produce *in vivo* mineralized matrix. Tissue Eng. Part A 17 (21–22), 2833–2843. 10.1089/ten.tea.2011.0062 21864161

[B108] Melke J. Midha S. Ghosh S. Ito K. Hofmann S. (2016). Silk fibroin as biomaterial for bone tissue engineering. Acta biomater. 31, 1–16. 10.1016/j.actbio.2015.09.005 26360593

[B109] Namini M. S. Ebrahimi-Barough S. Ai J. Jahromi H. K. mikaeiliagah E. Azami M. (2023). Tissue-engineered core--shell silk-fibroin/poly-l-lactic acid nerve guidance conduit containing encapsulated exosomes of human endometrial stem cells promotes peripheral nerve regeneration. ACS Biomaterials Sci. \& Eng. 9 (6), 3496–3511. 10.1021/acsbiomaterials.3c00157 37159418

[B110] Niu Y. Galluzzi M. Deng F. Zhao Z. Fu M. Su L. (2022). A biomimetic hyaluronic acid-silk fibroin nanofiber scaffold promoting regeneration of transected urothelium. Bioeng. \& Transl. Med. 7 (2), e10268. 10.1002/btm2.10268 35600655 PMC9115696

[B111] Pandey A. Yang T. S. Cheng S. L. Huang C. S. Brangule A. Kareiva A. (2021). A novel one-pot synthesis and characterization of silk fibroin/α-calcium sulfate hemihydrate for bone regeneration. Polymers 13 (12), 1996. 10.3390/polym13121996 34207134 PMC8235713

[B112] Park C. J. Ryoo J. Ki C. S. Kim J. W. Kim I. S. Bae D. G. (2018). Effect of molecular weight on the structure and mechanical properties of silk sericin gel, film, and sponge. Int. J. Biol. Macromol. 119, 821–832. 10.1016/j.ijbiomac.2018.08.006 30081122

[B113] Pham Q. P. Sharma U. Mikos A. G. (2006). Electrospinning of polymeric nanofibers for tissue engineering applications: a review. Tissue Eng. 12 (5), 1197–1211. 10.1089/ten.2006.12.1197 16771634

[B114] Plikk P. Målberg S. Albertsson A.-C. (2009). Design of resorbable porous tubular copolyester scaffolds for use in nerve regeneration. Biomacromolecules 10 (5), 1259–1264. 10.1021/bm900093r 19331401

[B115] Poza P. Pérez-Rigueiro J. Elices M. Llorca J. (2002). Fractographic analysis of silkworm and spider silk. Eng. Fract. Mech. 69 (9), 1035–1048. 10.1016/s0013-7944(01)00120-5

[B116] Raeisdasteh H. V. Davaran S. Ramazani A. Salehi R. (2017). Design and fabrication of porous biodegradable scaffolds: a strategy for tissue engineering. J. biomaterials Sci. 28 (16), 1797–1825. *polymer edition* . 10.1080/09205063.2017.1354674 28707508

[B117] Rajabi M. Firouzi M. Hassannejad Z. Haririan I. Zahedi P. (2018). Fabrication and characterization of electrospun laminin-functionalized silk fibroin/poly (ethylene oxide) nanofibrous scaffolds for peripheral nerve regeneration. J. Biomed. Mater. Res. Part B Appl. Biomaterials 106 (4), 1595–1604. 10.1002/jbm.b.33968 28805042

[B118] Ranger P. Senay A. Gratton G. R. Lacelle M. Delisle J. (2018). LARS synthetic ligaments for the acute management of 111 acute knee dislocations: effective surgical treatment for most ligaments. Knee Surg. Sports Traumatol. Arthrosc. 26, 3673–3681. 10.1007/s00167-018-4940-4 29691616

[B119] Rao F. Yuan Z. Li M. Yu F. Fang X. Jiang B. (2019). Expanded 3D nanofibre sponge scaffolds by gas-foaming technique enhance peripheral nerve regeneration. Artif. cells, nanomedicine, Biotechnol. 47 (1), 491–500. 10.1080/21691401.2018.1557669 30942090

[B120] Rao J. Cheng Y. Liu Y. Ye Z. Zhan B. Quan D. (2017). A multi-walled silk fibroin/silk sericin nerve conduit coated with poly (lactic-co-glycolic acid) sheath for peripheral nerve regeneration. Mater. Sci. Eng. C 73, 319–332. 10.1016/j.msec.2016.12.085 28183615

[B121] Rasheed T. Bilal M. Zhao Y. Raza A. Shah S. Z. H. Iqbal H. M. (2019). Physiochemical characteristics and bone/cartilage tissue engineering potentialities of protein-based macromolecules—a review. Int. J. Biol. Macromol. 121, 13–22. 10.1016/j.ijbiomac.2018.10.009 30291929

[B122] Ray W. Z. Mackinnon S. E. (2010). Management of nerve gaps: autografts, allografts, nerve transfers, and end-to-side neurorrhaphy. Exp. Neurol. 223 (1), 77–85. 10.1016/j.expneurol.2009.03.031 19348799 PMC2849924

[B123] Ribeiro V. P. Costa J. B. Carneiro S. M. Pina S. Veloso A. C. A. Reis R. L. (2022). Bioinspired silk fibroin-based composite grafts as bone tunnel fillers for anterior cruciate ligament reconstruction. Pharmaceutics 14 (4), 697. 10.3390/pharmaceutics14040697 35456531 PMC9029049

[B124] Ribeiro V. P. da Silva Morais A. Maia F. R. Canadas R. F. Costa J. B. Oliveira A. L. (2018). Combinatory approach for developing silk fibroin scaffolds for cartilage regeneration. Acta biomater. 72, 167–181. 10.1016/j.actbio.2018.03.047 29626700

[B125] Riccio M. Maraldi T. Pisciotta A. La Sala G. B. Ferrari A. Bruzzesi G. (2012). Fibroin scaffold repairs critical-size bone defects *in vivo* supported by human amniotic fluid and dental pulp stem cells. Tissue Eng. Part A 18 (9–10), 1006–1013. 10.1089/ten.tea.2011.0542 22166080

[B126] Rockwood D. N. Preda R. C. Yücel T. Wang X. Lovett M. L. Kaplan D. L. (2011). Materials fabrication from *Bombyx mori* silk fibroin. Nat. Protoc. 6 (10), 1612–1631. 10.1038/nprot.2011.379 21959241 PMC3808976

[B127] Rodbumrer P. Arthan D. Uyen U. Yuvaniyama J. Svasti J. Wongsaengchantra P. Y. (2012). Functional expression of a <italic&amp;gt;*Bombyx mori*&amp;lt;/italic&amp;gt; cocoonase: potential application for silk degumming. Acta Biochim. Biophys. Sin. 44 (12), 974–983. 10.1093/abbs/gms090 23169343

[B128] Saab M. Hildebrand F. Martel B. Blanchemain N. (2023). Osteoinductive bone morphogenic protein, collagen scaffold, calcium phosphate cement, and magnesium-based fixation enhance anterior cruciate ligament tendon graft to bone healing in animal models: a systematic review. Arthrosc. J. Arthrosc. \& Relat. Surg. 39 (2), 529–548.e9. 10.1016/j.arthro.2022.05.011 35714968

[B129] Sack B. S. Mauney J. R. Estrada C. R. (2016). Silk fibroin scaffolds for urologic tissue engineering. Curr. Urol. Rep. 17, 16–10. 10.1007/s11934-015-0567-x 26801192 PMC4856163

[B130] Sampaio K. A. Zyaykina N. Wozniak B. Tsukamoto J. Greyt W. D. Stevens C. V. (2015). Enzymatic degumming: degumming efficiency versus yield increase. Eur. J. Lipid Sci. Technol. 117 (1), 81–86. 10.1002/ejlt.201400218

[B131] Sen S. Basak P. Prasad Sinha B. Maurye P. Kumar Jaiswal K. Das P. (2020). Anti-inflammatory effect of epidermal growth factor conjugated silk fibroin immobilized polyurethane ameliorates diabetic burn wound healing. Int. J. Biol. Macromol. 143, 1009–1032. 10.1016/j.ijbiomac.2019.09.219 31647938

[B132] Sharafat-Vaziri A. Khorasani S. Darzi M. Saffarian Z. Alizadeh Z. Tahmasebi M. N. (2020). Safety and efficacy of engineered tissue composed of silk fibroin/collagen and autologous chondrocytes in two patients with cartilage defects: a pilot clinical trial study. Knee 27 (5), 1300–1309. 10.1016/j.knee.2020.06.015 33010742

[B133] Shen W. Chen X. Hu Y. Yin Z. Zhu T. Hu J. (2014). Long-term effects of knitted silk--collagen sponge scaffold on anterior cruciate ligament reconstruction and osteoarthritis prevention. Biomaterials 35 (28), 8154–8163. 10.1016/j.biomaterials.2014.06.019 24974007

[B134] Shi W. Sun M. Hu X. Ren B. Cheng J. Li C. (2017). Structurally and functionally optimized silk-fibroin--gelatin scaffold using 3D printing to repair cartilage injury *in vitro* and *in vivo* . Adv. Mater. 29 (29), 1701089. 10.1002/adma.201701089 28585319

[B135] Singh B. N. Pramanik K. (2018). Fabrication and evaluation of non-mulberry silk fibroin fiber reinforced chitosan based porous composite scaffold for cartilage tissue engineering. Tissue Cell 55, 83–90. 10.1016/j.tice.2018.10.003 30503064

[B136] Smith C. Ajuied A. Wong F. Norris M. Back D. Davies A. (2014). The use of the ligament augmentation and reconstruction system (LARS) for posterior cruciate reconstruction. Arthrosc. J. Arthrosc. \& Relat. Surg. 30 (1), 111–120. 10.1016/j.arthro.2013.09.081 24290790

[B137] Sothornvit R. Chollakup R. Suwanruji P. (2010). Extracted sericin from silk waste for film formation. Songklanakarin J. Sci. \& Technol. 32 (1).

[B138] Srisawasdi T. Petcharoen K. Sirivat A. Jamieson A. M. (2015). Electromechanical response of silk fibroin hydrogel and conductive polycarbazole/silk fibroin hydrogel composites as actuator material. Mater. Sci. Eng. C 56, 1–8. 10.1016/j.msec.2015.06.005 26249559

[B139] Suesca E. Dias A. Braga M. de Sousa H. Fontanilla M. (2017). Multifactor analysis on the effect of collagen concentration, cross-linking and fiber/pore orientation on chemical, microstructural, mechanical and biological properties of collagen type I scaffolds. Mater. Sci. Eng. C 77, 333–341. 10.1016/j.msec.2017.03.243 28532037

[B140] Sultan M. T. (2018). Silk fibroin in wound healing process. Novel Biomaterials for Regenerative Medicine, 115–126. 10.1007/978-981-13-0947-2_7 30357686

[B141] Sun B. Wu T. Wang J. Li D. Wang J. Gao Q. (2016a). Polypyrrole-coated poly(l-lactic acid-co-ε-caprolactone)/silk fibroin nanofibrous membranes promoting neural cell proliferation and differentiation with electrical stimulation. J. Mater. Chem. B 4 (41), 6670–6679. 10.1039/c6tb01710j 32263522

[B142] Sun J. Li L. Xing F. Yang Y. Gong M. Liu G. (2021a). Graphene oxide-modified silk fibroin/nanohydroxyapatite scaffold loaded with urine-derived stem cells for immunomodulation and bone regeneration. Stem Cell Res. \& Ther. 12, 591–620. 10.1186/s13287-021-02634-w 34863288 PMC8642892

[B143] Sun L. Parker S. T. Syoji D. Wang X. Lewis J. A. Kaplan D. L. (2012). Direct-write assembly of 3D silk/hydroxyapatite scaffolds for bone co-cultures. Adv. Healthc. Mater. 1 (6), 729–735. 10.1002/adhm.201200057 23184824 PMC3739986

[B144] Sun W. Gregory D. A. Tomeh M. A. Zhao X. (2021b). Silk fibroin as a functional biomaterial for tissue engineering. Int. J. Mol. Sci. 22 (3), 1499. 10.3390/ijms22031499 33540895 PMC7867316

[B145] Sun X. Hao Y. Wang Q. Dong F. Wang J. (2016b). Cell growth and proliferation on the interface of a silk fabric tubular scaffold. Text. Res. J. 86 (20), 2193–2201. 10.1177/0040517515622146

[B146] Tanaka T. Tanaka R. Ogawa Y. Takagi Y. Asakura T. (2020). Development of small-diameter polyester vascular grafts coated with silk fibroin sponge. Organogenesis 16 (1), 1–13. 10.1080/15476278.2019.1686295 31679437 PMC7051131

[B147] Tanaka T. Tanaka R. Ogawa Y. Takagi Y. Sata M. Asakura T. (2021). Evaluation of small-diameter silk vascular grafts implanted in dogs. JTCVS open 6, 148–156. 10.1016/j.xjon.2021.02.008 36003556 PMC9390453

[B148] Tang X. Xue C. Wang Y. Ding F. Yang Y. Gu X. (2012). Bridging peripheral nerve defects with a tissue engineered nerve graft composed of an *in vitro* cultured nerve equivalent and a silk fibroin-based scaffold. Biomaterials 33 (15), 3860–3867. 10.1016/j.biomaterials.2012.02.008 22364696

[B149] Tariq M. Tahir H. M. Butt S. A. Ali S. Ahmad A. B. Raza C. (2021). Silk derived formulations for accelerated wound healing in diabetic mice. PeerJ 9, e10232. 10.7717/peerj.10232 33510964 PMC7798629

[B150] Thurber A. E. Omenetto F. G. Kaplan D. L. (2015). *In vivo* bioresponses to silk proteins. Biomaterials 71, 145–157. 10.1016/j.biomaterials.2015.08.039 26322725 PMC4573254

[B151] Türkkan S. Pazarçeviren A. E. Keskin D. Machin N. E. Duygulu Ö. Tezcaner A. (2017). Nanosized CaP-silk fibroin-PCL-PEG-PCL/PCL based bilayer membranes for guided bone regeneration. Mater. Sci. Eng. C 80, 484–493. 10.1016/j.msec.2017.06.016 28866191

[B152] Ullah S. Chen X. (2020). Fabrication, applications and challenges of natural biomaterials in tissue engineering. Appl. Mater. Today 20, 100656. 10.1016/j.apmt.2020.100656

[B153] Unajak S. Aroonluke S. Promboon A. (2015). An active recombinant cocoonase from the silkworm *Bombyx mori*: bleaching, degumming and sericin degrading activities. J. Sci. Food Agric. 95 (6), 1179–1189. 10.1002/jsfa.6806 25042939

[B154] Vepari C. Kaplan D. L. (2007). Silk as a biomaterial. Prog. Polym. Sci. 32 (8–9), 991–1007. 10.1016/j.progpolymsci.2007.05.013 19543442 PMC2699289

[B155] Vetsch J. R. Paulsen S. J. Müller R. Hofmann S. (2015). Effect of fetal bovine serum on mineralization in silk fibroin scaffolds. Acta biomater. 13, 277–285. 10.1016/j.actbio.2014.11.025 25463486

[B156] Vishwanath V. Pramanik K. Biswas A. (2016). Optimization and evaluation of silk fibroin-chitosan freeze-dried porous scaffolds for cartilage tissue engineering application. J. Biomaterials Sci. Polym. Ed. 27 (7), 657–674. 10.1080/09205063.2016.1148303 26830046

[B157] von Byern J. Chandler P. Merritt D. Adlassnig W. Stringer I. Meyer-Rochow V. B. (2019). Biomechanical properties of fishing lines of the glowworm Arachnocampa luminosa (Diptera; Keroplatidae). Sci. Rep. 9 (1), 3082. 10.1038/s41598-019-39098-1 30816149 PMC6395680

[B158] Wang H.-Y. Wei Z.-G. Zhang Y.-Q. (2020a). Dissolution and regeneration of silk from silkworm *Bombyx mori* in ionic liquids and its application to medical biomaterials. Int. J. Biol. Macromol. 143, 594–601. 10.1016/j.ijbiomac.2019.12.066 31836392

[B159] Wang Q. Han G. Yan S. Zhang Q. (2019a). 3D printing of silk fibroin for biomedical applications. Materials 12 (3), 504. 10.3390/ma12030504 30736388 PMC6384667

[B160] Wang Y. Yu H. Liu H. Fan Y. (2020b). Double coating of graphene oxide--polypyrrole on silk fibroin scaffolds for neural tissue engineering. J. Bioact. Compatible Polym. 35 (3), 216–227. 10.1177/0883911520913905

[B161] Wang Z. Yang H. Li W. Li C. (2019b). Effect of silk degumming on the structure and properties of silk fibroin. J. Text. Inst. 110 (1), 134–140. 10.1080/00405000.2018.1473074

[B162] Wohlrab S. Müller S. Schmidt A. Neubauer S. Kessler H. Leal-Egaña A. (2012). Cell adhesion and proliferation on RGD-modified recombinant spider silk proteins. Biomaterials 33 (28), 6650–6659. 10.1016/j.biomaterials.2012.05.069 22727466

[B163] Woloszyk A. Buschmann J. Waschkies C. Stadlinger B. Mitsiadis T. A. (2016). Human dental pulp stem cells and gingival fibroblasts seeded into silk fibroin scaffolds have the same ability in attracting vessels. Front. physiology 7, 196818. 10.3389/fphys.2016.00140 PMC4835714 27148078

[B164] Wöltje M. Böbel M. Bienert M. Neuss S. Aibibu D. Cherif C. (2018). Functionalized silk fibers from transgenic silkworms for wound healing applications: surface presentation of bioactive epidermal growth factor. J. Biomed. Mater. Res. Part A 106 (10), 2643–2652. 10.1002/jbm.a.36458 29790257

[B165] Wu T. Chen Y. Liu W. Tong K. L. Suen C. W. W. Huang S. (2020). Ginsenoside Rb1/TGF-β1 loaded biodegradable silk fibroin-gelatin porous scaffolds for inflammation inhibition and cartilage regeneration. Mater. Sci. Eng. C 111, 110757. 10.1016/j.msec.2020.110757 32279738

[B166] Wu Y. Zhou L. Li Y. Lou X. (2022). Osteoblast-derived extracellular matrix coated PLLA/silk fibroin composite nanofibers promote osteogenic differentiation of bone mesenchymal stem cells. J. Biomed. Mater. Res. Part A 110 (3), 525–534. 10.1002/jbm.a.37302 34494712

[B167] Wuttisiriboon K. Tippayawat P. Daduang J. Limpaiboon T. (2023). Three-dimensional silk fibroin-gelatin/chondroitin sulfate/hyaluronic acid--aloe vera scaffold supports *in vitro* chondrogenesis of bone marrow mesenchymal stem cells and reduces inflammatory effect. J. Biomed. Mater. Res. Part B Appl. Biomaterials 111 (8), 1557–1570. 10.1002/jbm.b.35254 36988305

[B168] Xiaohalati X. Wang J. Su Q. Wang Y. Liu J. Li H. (2024). A materiobiology-inspired sericin nerve guidance conduit extensively activates regeneration-associated genes of Schwann cells for long-gap peripheral nerve repair. Chem. Eng. J. 483, 149235. 10.1016/j.cej.2024.149235

[B169] Xie C. Ye J. Liang R. Yao X. Wu X. Koh Y. (2021). Advanced strategies of biomimetic tissue-engineered grafts for bone regeneration. Adv. Healthc. Mater. 10 (14), 2100408. 10.1002/adhm.202100408 33949147

[B170] Xie S.-Y. Peng L. H. Shan Y. H. Niu J. Xiong J. Gao J. Q. (2016). Adult stem cells seeded on electrospinning silk fibroin nanofiberous scaffold enhance wound repair and regeneration. J. Nanosci. Nanotechnol. 16 (6), 5498–5505. 10.1166/jnn.2016.11730 27427589

[B171] Xiong S. Zhang X. Lu P. Wu Y. Wang Q. Sun H. (2017). A gelatin-sulfonated silk composite scaffold based on 3D printing technology enhances skin regeneration by stimulating epidermal growth and dermal neovascularization. Sci. Rep. 7 (1), 4288. 10.1038/s41598-017-04149-y 28655891 PMC5487355

[B172] Xu W.-P. Zhang W. Asrican R. Kim H. J. Kaplan D. L. Yelick P. C. (2008). Accurately shaped tooth bud cell--derived mineralized tissue formation on silk scaffolds. Tissue Eng. Part A 14 (4), 549–557. 10.1089/tea.2007.0227 18352829

[B173] Xu Y. Xia D. Han J. Yuan S. Lin H. Zhao C. (2017). Design and fabrication of porous chitosan scaffolds with tunable structures and mechanical properties. Carbohydr. Polym. 177, 210–216. 10.1016/j.carbpol.2017.08.069 28962760

[B174] Xue C. Zhu H. Tan D. Ren H. Gu X. Zhao Y. (2018). Electrospun silk fibroin-based neural scaffold for bridging a long sciatic nerve gap in dogs. J. tissue Eng. Regen. Med. 12 (2), e1143–e1153. 10.1002/term.2449 28485084

[B175] Yagi T. Sato M. Nakazawa Y. Tanaka K. Sata M. Itoh K. (2011). Preparation of double-raschel knitted silk vascular grafts and evaluation of short-term function in a rat abdominal aorta. J. Artif. Organs 14, 89–99. 10.1007/s10047-011-0554-z 21344164

[B176] Yamamoto S. Okamoto H. Haga M. Shigematsu K. Miyata T. Watanabe T. (2016). Rapid endothelialization and thin luminal layers in vascular grafts using silk fibroin. J. Mater. Chem. B 4 (5), 938–946. 10.1039/c5tb02528a 32263167

[B177] Yan S. Li Y. Jiang Y. C. Xu Y. Wang D. Zhang X. (2021). Expanded polytetrafluoroethylene/silk fibroin/salicin vascular graft fabrication for improved endothelialization and anticoagulation. Appl. Surf. Sci. 542, 148610. 10.1016/j.apsusc.2020.148610

[B178] Yang L. Wang X. Xiong M. Liu X. Luo S. Luo J. (2024). Electrospun silk fibroin/fibrin vascular scaffold with superior mechanical properties and biocompatibility for applications in tissue engineering. Sci. Rep. 14 (1), 3942. 10.1038/s41598-024-54638-0 38365964 PMC10873321

[B179] Yang Q. Teng B. H. Wang L. N. Li K. Xu C. Ma X. L. (2017). Silk fibroin/cartilage extracellular matrix scaffolds with sequential delivery of TGF-&beta;3 for chondrogenic differentiation of adipose-derived stem cells. Int. J. nanomedicine 12, 6721–6733. 10.2147/ijn.s141888 28932116 PMC5600265

[B180] Yang Y. J. Kwon Y. Choi B. H. Jung D. Seo J. H. Lee K. H. (2014). Multifunctional adhesive silk fibroin with blending of RGD-bioconjugated mussel adhesive protein. Biomacromolecules 15 (4), 1390–1398. 10.1021/bm500001n 24601579

[B181] Zafar B. Mottaghitalab F. Shahosseini Z. Negahdari B. Farokhi M. (2020). Silk fibroin/alumina nanoparticle scaffold using for osteogenic differentiation of rabbit adipose-derived stem cells. Materialia 9, 100518. 10.1016/j.mtla.2019.100518

[B182] Zhang J. Wang L. Xu C. Cao Y. Liu S. Reis R. L. (2024). Transparent silk fibroin film-facilitated infected-wound healing through antibacterial, improved fibroblast adhesion and immune modulation. J. Mater. Chem. B 12 (2), 475–488. 10.1039/d3tb02146g 38099432

[B183] Zhang W. Ahluwalia I. P. Literman R. Kaplan D. L. Yelick P. C. (2011). Human dental pulp progenitor cell behavior on aqueous and hexafluoroisopropanol based silk scaffolds. J. Biomed. Mater. Res. Part A 97 (4), 414–422. 10.1002/jbm.a.33062 PMC3126627 21484985

[B184] Zhang X. Baughman C. B. Kaplan D. L. (2008). *In vitro* evaluation of electrospun silk fibroin scaffolds for vascular cell growth. Biomaterials 29 (14), 2217–2227. 10.1016/j.biomaterials.2008.01.022 18279952 PMC2698960

[B185] Zhang X. Reagan M. R. Kaplan D. L. (2009). Electrospun silk biomaterial scaffolds for regenerative medicine. Adv. drug Deliv. Rev. 61 (12), 988–1006. 10.1016/j.addr.2009.07.005 19643154 PMC2774469

[B186] Zhao H.-P. Feng X.-Q. Shi H.-J. (2007). Variability in mechanical properties of *Bombyx mori* silk. Mater. Sci. Eng. C 27 (4), 675–683. 10.1016/j.msec.2006.06.031

[B187] Zhi Y. Jiang J. Zhang P. Chen S. (2019). Silk enhances the ligamentization of the polyethylene terephthalate artificial ligament in a canine anterior cruciate ligament reconstruction model. Artif. Organs 43 (6), E94–E108. 10.1111/aor.13389 30412273

